# Natural Antioxidant Evaluation: A Review of Detection Methods

**DOI:** 10.3390/molecules27113563

**Published:** 2022-06-01

**Authors:** Jenifer da Silva Mendonça, Rita de Cássia Avellaneda Guimarães, Verônica Assalin Zorgetto-Pinheiro, Carolina Di Pietro Fernandes, Gabriela Marcelino, Danielle Bogo, Karine de Cássia Freitas, Priscila Aiko Hiane, Elaine Silva de Pádua Melo, Marcelo Luiz Brandão Vilela, Valter Aragão do Nascimento

**Affiliations:** 1 Graduate Program in Health and Development in the Central-West Region of Brazil, Federal University of Mato Grosso do Sul, Campo Grande 79070-900, Brazil; jenifersilvamdc@gmail.com (J.d.S.M.); rita.guimaraes@ufms.br (R.d.C.A.G.); veronica.azp@outlook.com (V.A.Z.-P.); gabi19ac@gmail.com (G.M.); daniellebogo@hotmail.com (D.B.); kcfreitas@gmail.com (K.d.C.F.); priscila.hiane@ufms.br (P.A.H.); elaine.melo@ufms.br (E.S.d.P.M.); 2 Group of Spectroscopy and Bioinformatics Applied Biodiversity and Health (GEBABS), Federal University of Mato Grosso do Sul, Campo Grande 79070-900, Brazil; carolfernands2@gmail.com; 3 School of Medicine, Federal University of Mato Grosso do Sul, Campo Grande 79070-900, Brazil; marcelo.vilela@ufms.br

**Keywords:** antioxidant, bioactive compounds, secondary metabolites, antioxidant assay, in vitro models, in vivo models

## Abstract

Antioxidants have drawn the attention of the scientific community due to being related to the prevention of various degenerative diseases. The antioxidant capacity has been extensively studied in vitro, and different methods have been used to assess its activity. However, the main issues related to studying natural antioxidants are evaluating whether these antioxidants demonstrate a key role in the biological system and assessing their bioavailability in the organism. The majority of outcomes in the literature are controversial due to a lack of method standardization and their proper application. Therefore, this study aims to compile the main issues concerning the natural antioxidant field of study, comparing the most common in vitro methods to evaluate the antioxidant activity of natural compounds, demonstrating the antioxidant activity in biological systems and the role of the main antioxidant enzymes of redox cellular signaling and explaining how the bioavailability of bioactive compounds is evaluated in animal models and human clinical trials.

## 1. Introduction

The term “antioxidant” refers to substances or molecules that are capable of delaying or even preventing the irreversible damage of other substances/macromolecules due to some metabolites’ instability present in a living system and thus promoting health benefits, since oxidative stress is the root of several pathophysiological processes. Beyond a comprehensive definition, antioxidant activity tests require different approaches, since different classes of substances may present such activity, ranging from the well-known vitamin C to the not-so-recently but continuously described peptides derived from animal or vegetable sources. For instance, measuring antioxidant activity implies measuring the reaction rate or how antioxidants may affect the autoxidation rate of the substrate which they are known to protect [1]. Exogenous natural antioxidants may be considered as bioactive compounds and are specially derived from food and medicinal plants, such as fruits, vegetables, cereals, spices and traditional herbs [2,3,4,5,6]. Natural antioxidants present in foods, such as phenolic phytochemicals, seem to provide metabolic benefits and are associated with a lower risk of developing several health problems [7]. In addition, the protective effects of antioxidants present in fruits and vegetables are related to three main groups: carotenoids, phenolic compounds and vitamins [8]. 

The supply of antioxidants acts on neutralizing reactive oxygen species (ROS), which are produced in the system during physiological processes [9]. Singlet oxygen, superoxide, hydrogen peroxide (H_2_O_2_), peroxynitrite, hydroxyl and peroxyl radicals are examples of ROS, and they are ubiquitous and potentially harmful to valuable biomolecules [10,11]. Excessive and uncontrolled ROS production is called oxidative stress, which could lead to damage, such as alterations in cell function which are linked to several conditions such as chronic inflammation, asthma, neurodegenerative and cardiovascular diseases, senescence and cancer [12,13].

The importance of the increase in antioxidant consumption through food has attracted much attention for the development of measurement techniques assessing foods’ antioxidant content [14,15]. The concentration of specific antioxidants cannot predict the antioxidant capacity in samples, which depends on a variety of compounds, some of which, after their metabolization, might not be identified [16]. Antioxidants act synergistically, both in vitro and in vivo [17], and so the determination of isolated antioxidant compounds could result in under- or overestimation of the antioxidant activity.

Since the antioxidant capacity cannot be measured directly, assessment methods help to evaluate the effects of antioxidants to control the extent of oxidation. The oxidation reaction features a substrate, an oxidant and an initiator, intermediates, and final products. The assessment of the antioxidant capacity can be performed by the measurement of any of these items [18]. Several methods are used to examine the antioxidant properties of samples, which may come from fruits, vegetables, plant extracts or commercial antioxidants. Basically, two types of approaches are used: hydrogen atom transfer (HAT) based-methods and electron transfer (ET) based-methods. Nonetheless, assays that represent total antioxidant capacity (TAC), such as the 2,2′-azino-bis-3-ethylbenzthiazoline-6-sulphonic acid (ABTS) assay, generally use measurement methods that are non-competitive ET and mixed-mode (ET/HAT) [19].

TAC methods are used to determine both the antioxidant capacity of food and the food components, such as plants, extracts, processed foods, beverages and isolated compounds. The evaluation of TAC in foods can be carried out in chemical or biological oxidizable substances (plasma or cultured cells). Likewise, TAC methods are also used to assess antioxidant activity in tissue and body fluids to associate TAC results with specific conditions, e.g., altered redox status, oxidative stress and disease states, or treatments, e.g., diet, supplementation and pharmacological treatment [20].

In addition to the known nutritional functionality of antioxidant compounds, it is important to consider that most of the antioxidant phytochemicals derived from food intake can be metabolized and conjugated during the absorption process [21,22]. Therefore, it is necessary to extend the understanding of metabolism, absorption, bioavailability and the mechanism of antioxidants and their bioactive properties [7]. Accumulating evidence indicates that the beneficial properties of food components with antioxidant capacity using in vitro models occur through very specific mechanisms rather than direct antioxidant effects [23].

Considering that some studies have focused on the development of innovative therapeutic targets from natural compounds, the aim of this review is to compare the most important antioxidant activity in in vitro methods and to evaluate biological antioxidant activity through enzymes in in vivo models related to cellular redox signaling, along with the bioavailability of some antioxidant phytochemicals derived from food intake.

## 2. Natural Antioxidants

### 2.1. Carotenoids

Carotenoids are mainly C40 lipophilic isoprenoids, and are the second most abundant naturally occurring pigments on Earth, with more than 700 members. Carotenoids range from colorless to yellow and orange to red, conferring color to fruits and vegetables [24,25]. They occur in all photosynthetic organisms, and can also be found in autotrophic bacteria [26,27,28]. Although animals and humans do not synthesize carotenoids, it is present in their blood and tissues as precursors of retinol (vitamin A) [26]. Usually, carotenoids are located inside cell membranes as highly lipophilic molecules. While strict hydrocarbons, such as lycopene or β-carotene, are arranged exclusively in the inner part of the lipid bilayer, molecules with a more polar configuration containing oxygen atoms attached (like lutein or zeaxanthin) are oriented roughly perpendicular to the membrane surface with their hydrophilic parts oriented to the aqueous environment [29,30].

Oxidative stress is related to many diseases, such as obesity and metabolic syndrome [31], atherosclerosis [32], Parkinson’s disease [33], and Alzheimer’s disease [34]. On the other hand, carotenoid intake has been associated with a reduction in the risk of developing chronic diseases such as cancer, cardiovascular diseases, cataracts and macular degeneration [35].

Carotenoids are characterized by an extended conjugated π-electron system that aids in the stabilization of unpaired electrons after radical quenching [36]. They are also able to deactivate peroxyl radicals by reacting with them to form resonance stabilized carbon centered radical adducts [37], and they are known as efficient physical quenchers of oxygen in both in vitro and in vivo models [38,39,40]. Generally, the O_2_ radical deactivation by carotenoids occurs based on the conversion of an excess of energy to heat via the carotenoid’s lowest excited state. Thus, the possible damaging effects of excited carotenoids can be disregarded due to their low energy and short lifetime [27].

In recent years, concentrated efforts have emerged to develop improved extraction methods for carotenoids. However, for the extraction of some complex food matrices, the efficiency remains low, as there are several physical and chemical barriers present in the food that prevent mass transfer during extraction [41].

The presence of molecules with varying levels of polarity also makes extraction complex. Furthermore, the oxidative property of carotenoids limits exposure to light, heat, sudden pH variations and long extraction times. Due to their hydrophobic nature, carotenoids are conventionally extracted using organic solvents [42].

Typically, nonpolar solvents such as hexane, petroleum ether or tetrahydrofuran (THF) are a good choice for extracting non-polar carotenoids or esterified xanthophylls, while more polar solvents such as acetone, ethanol and ethyl acetate are more suitable for the extraction of polar carotenoids [43].

The different methods used for the extraction of carotenoids from natural sources can be classified into the following categories: extraction of liquid atmosphere using Soxhlet; maceration; microwave (microwave-assisted extraction); ultrasound (ultrasound-assisted extraction); accelerated solvent extraction, also known as pressurized liquid extraction (PLE); pulsed electric field assisted extraction; supercritical fluid extraction based on the use of CO_2_ as a solvent; and enzyme-assisted extraction (EAE). All these extraction methods differ in the mode of cell disintegration, temperature used, and pressure applied for the extraction of carotenoids [44].

Carotenoids can be extracted using organic solvents such as acetone, chloroform, hexane, isopropanol, methanol, methylene chloride and diethyl ether. A wide variety of solvent combinations can also be used, which provides a synergistic effect in extraction and consequently greater extraction effectiveness due to different polarities. Choosing the appropriate solvent or solvent combination is one of the most important factors for the efficient extraction of carotenoids [45].

Choosing the appropriate solvent is not simple, as functional group polarity as well as chain length, moisture content, sample matrix and its components play important roles in the extraction. In addition, lycopene and β-carotene are highly lipophilic nonpolar carotenoids due to their structure of conjugated hydrocarbons without polar functional groups [46].

Acetone and hexane are often selected for the extraction of polar and nonpolar carotenoids. On the other hand, an acetone/ethanol/hexane mixture is more often applied for the simultaneous extraction of more polar and less polar carotenoids. Due to their miscible properties in water, acetone and ethanol are more efficient for extracting carotenoids from plant material containing a large amount of moisture [47].

Most solvents used for the extraction of carotenoids, including ethanol, hexane and acetone, cause environmental and human health risks (acute and chronic toxicity) [48]. However, based on environmental issues and, above all, occupational safety, ethanol and acetone are preferred solvents compared to hexane, diethyl ether, dichloromethane and chloroform [49]. To improve sustainability, “green solvents” and ecologically sound ionic liquids can be exploited for the extraction of carotenoids and other bioactive compounds [50].

### 2.2. Vitamin E

The term vitamin E is used to describe eight lipophilic, naturally occurring compounds that include four tocopherols and four tocotrienols. All vitamin E forms have a chromanol ring and a 16-carbon phytyl-like side chain. Depending on the nature of the isoprenoid chain, it is possible to distinguish tocopherols with a saturated phytyl chain and tocotrienols with an unsaturated geranylgeranyl chain having three double bonds [51,52,53].

Animals and humans cannot synthesize vitamin E, which is acquired through the gestation of vegetables in food (especially tocopherols). γ-tocopherol is the most prevalent in seeds and derived products [54,55], while α-tocopherol is the predominant form of vitamin E in plant tissues. The low intake of vitamin E is associated with its deficiency and ataxia [56]. Vitamin E acts as a lipid phase chain-breaking antioxidant that appears effective in improving health outcomes in clinical trials, with diminished risk of cancer and cardiovascular diseases [57]. As part of cell membranes and lipoproteins, due to its apolar property, vitamin E protects lipids from oxidation [58]. Moreover, vitamin E fights against lipid peroxidation of cell membranes and can stop the radical chain by forming a low-reactivity derivative unable to attack lipid substrates [59].

All tocopherols and tocotrienols are potent antioxidants with lipoperoxyl radical-scavenging activities [51] through hydrogen donation from the phenolic group on the chromanol ring [60,61]. Natural vitamin E forms possessing an unsubstituted 5-position, such as γ-tocopherol, can trap electrophiles, including reactive nitrogen species (NOS), which are found to be enhanced during inflammation. Vitamin E forms with a methyl group at 5-position, such as α-tocopherol, do not trap electrophiles [54]. Therefore, γ-tocopherol is shown to be better than α-tocopherol in detoxifying NO_2_ and peroxynitrite via 5-nitro-γ-tocopherol formation [62,63,64].

Traditional solvent extraction methods after saponification have been widely used in extracting vitamin E compounds from different types of samples, including food and feed, tissue and biological fluids [65]. The purpose of saponification is to hydrolyze esters linked to glycerides, phospholipids and sterols, as well as to destroy pigments and disrupt the sample matrix to release vitamin E compounds. However, saponification can affect the analysis of vitamin E in biological samples due to the degradation results of these compounds, which in turn are not very stable under alkaline conditions [66].

Ultrasound-assisted extraction has been used in the analysis of pesticides or organic contaminants in the soil, animal tissue and food packaging materials. Furthermore, it has been used in the extraction of oils and phytochemicals [67]. Ultrasound-assisted extraction can increase the yield of target compound extraction in sample preparation. That is, the frequency of ultrasound can break the micelle or sample matrix to facilitate solvent access to hydrophobic compounds. This is in contrast with saponification breakdown under alkaline conditions, as there would be no chemical involvement in the ultrasound-assisted extraction, which could prevent a possible chemical degradation of the target components [68]. Furthermore, the ultrasound, when interacting with the extraction solvent, increases the contact between the solvent and the target compounds, making the extraction more efficient. Ultrasound can be applied to many other processes, including intracellular metabolite extraction and microbial inactivation. The greatest impact of ultrasound in liquid media is attributed to acoustic cavitation, leading to cell rupture, which enhances the mass transfer of the extractors [69].

The ultrasonic power, intensity, temperature and density of the mixture (solvent/sample ratio) are important factors to be optimized for efficient extraction of metabolites. The use of an optimized ultrasound intensity range is a crucial parameter to obtain a higher extraction yield. Above the optimal range, increased ultrasound intensity can lead to the formation and accumulation of OH^-^ and H^+^ radicals during the cavitation process, which can lead to the significant degradation of antioxidant compounds, including carotenoids [70].

### 2.3. Ascorbic Acid

Ascorbic acid, also known as vitamin C, is a ketolactone with a molecular weight of 176.1 g/mol and has polar properties, being soluble in water and considered a very important component for human health [71,72]. Different from humans, most mammals are able to synthesize ascorbic acid from glucuronic acid or galactonic acid derived from glucose [71]. As a non-enzymatic antioxidant, vitamin C works by interrupting free radical chain reactions [37]. Ascorbic acid is endowed with enhanced antioxidant activity, being promptly oxidized to dehydroascorbic acid, which suffers irreversible hydrolysis to 2,3-diketo-L-gulonic acid with subsequent decarboxylation, resulting in carbon dioxide and pentose phosphate cycle components, or oxalic acid and threonic acid. Ascorbic acid is involved in maintaining vascular and connective tissue integrity in iron absorption and collagen biosynthesis, neuroprotection, hematopoiesis and leucocyte functioning [13,73,74]. Ascorbic acid in the brain plays an essential role, being a cofactor of dopamine beta-hydroxylase, and thus being part of catecholamine biosynthesis. Additionally, it protects membrane phospholipids from peroxidative damage and is an efficient free-radical scavenger in the brain [74,75]. Ascorbic acid accelerates hydroxylation reactions in several biosynthetic pathways. During many of these reactions, ascorbic acid directly or indirectly can provide electrons to enzymes that require prosthetic metal ions in a reduced form to function with full enzymatic activity [76]. Although ascorbic acid is not a direct scavenger of lipophilic radicals, it acts in synergy with tocopherols in lipid peroxide radical removal by regenerating vitamin E in combination with reduced glutathione (GSH) or with compounds capable of donating reducing equivalents [37,77,78,79].

### 2.4. Phenolic Compounds

The course of different diseases can lead to increased oxidative stress, which triggers the development of metabolic disorders such as the increased production of reactive oxygen species (ROS) and the glycosylation of non-enzymatic proteins [80]. All these factors can damage DNA molecules, lipids and proteins, leading to the development of diseases such as rheumatism, inflammatory bowel diseases, coronary heart disease [81], cancer, diabetes and degenerative diseases [82]. In this context, medicinal plants have been widely used over time to treat different diseases [83], as they have natural phytochemicals that can be present in leaves, stems, fruits, flowers, roots and bark that represent an important source of antioxidant compounds such as flavonoids, phenolic acids, tannins, anthocyanins and other phenolic compounds [81,84,85], which can treat or prevent oxidation effects; thus, medicinal plants arouse great interest in the food and pharmaceutical industry [86].

There are a great variety of phenolic structures present in plants. These compounds grant plants protection against any damage that microorganisms, pests and predators may cause. They influence the nutritional and sensorial quality of food, conferring colors, textures and astringency [87]. Phenolic compounds are classified as secondary bioactive metabolites, possessing at least one aromatic ring linked to one or more hydroxyl groups, with a simple structure to high molecular weight complex polymers [88]. They are divided into flavonoids and non-flavonoids and their subclasses, based on the number of linked hydroxyls and to structural elements that connect benzene rings [89].

The flavonoid group in nature contributes to the majority of phenols, representing up to 50% of all phenols [90] and, until now, over 9000 flavonoids have been reported [91]. They are derived from aromatic amino acids, phenylalanine and tyrosine, containing 15 carbon chains with two benzene rings linked by a heterocyclic pyrene ring [90]. Flavonoids occur as aglycones, glycosides and methylated derivatives, where aglycones hold the most basic structure of all; they are synthesized by acetate and shikimic acid pathways [92]. The main subclasses of flavonoids are flavonols, flavones, flavan-3-ols, anthocyanins, flavonones and isoflavones, and they can be differentiated by small variations in the structure of the carbon chain [90,93].

Flavonols are the most prevalent in the plant kingdom, with the exceptions of algae and fungi. The main components are kaempferol, quercetin, myricetin and isorhamnetin. They possess a ketone group in their structure and hydroxyl in position 3 of their C- ring [90,94]. Conversely, flavones are less present in the plant kingdom, except for in some herbs. Some citrus species contain hesperidin and naringenin. Regarding the chemical structure, flavan-3-ols are the most complex of flavonoids, varying from simple monomers, such as catechins, to complex structures such as condensed tannins. The hydroxyl group of those compounds is always interacting with the position 3 of the C-aromatic ring [90].

Anthocyanins are present in different plants and are responsible for red, blue and purple colors. These pigments are employed in the food industry to promote color without using synthetic food coloring [95]. These colors depend on pH levels, methylation and acylation of hydroxyl groups in the rings A and B. The most common types are cyanidin, peonidin, petunidin, malvidin, delphinidin and pelargonidin, which are differentiated by the number of hydroxyl groups, the nature and the number of sugars that these groups are linked to and their position, and being monosaccharides, the most likely sugar in these positions [90].

Flavonoids are mainly present in citrus fruits. This group is associated with health benefits due to its ability to quench free radicals [90,91]. The bond, in flavonone structure, is found at positions 2 and 3 in the aromatic ring [90,94]. Additionally, isoflavones, also known as phytoestrogens, are responsible for ovulation blockage, especially in animals that feed directly from the source with a high concentration of this compound. Isoflavone intake by humans could aid in prostate and breast cancer prevention due its hormone-like property. Isoflavone can be found in a limited number of plants, such as soybean. These compounds differ from other flavones because their B-aromatic ring is linked to the C-ring by the carbon 3 [90].

Phenolic acids, tannins, stilbenes and lignans represent the non-flavonoid group [89]. Phenolic acids are phenols that have carboxylic acid function and are divided into hydroxybenzoic and hydroxycinnamic acids. Hydroxybenzoic acid possesses a C6–C1 structure in its aromatic rings and is derived from dihydrobenzoic acid. Its antioxidant activity is dependent on the hydroxyl position in the ring. There are five types of phenolic acids: gallic, p-hydroxybenzoic, procatequic, vanillic, and syringic acids. Hydroxycinnamic acids hold the C6–C3 structure and occur in various conjugated forms, such as hydroxy acid esters. Caffeic acid is present in several foods, is responsible for blocking leukotriene synthesis and is involved in immunoregulatory diseases. Other compounds of this family include ferulic and synaptic acids [96]. The antioxidant activity of this group occurs via hydrogen electron donation to free radicals [97]. Stilbenes have a core of 1,2-diphenylethylene with hydroxyl groups in their aromatic rings and are produced by plants as a response to sickness or injuries, with trans-resveratrol the best-known dietetic form. Trans-resveratrol is present in red wine and is able to inhibit and slow down a number of pathological conditions, such as cardiovascular diseases and some types of cancer [90,96].

Lignans are precursors of phytoestrogens, found in seeds, vegetable oils, cereals, fruits and vegetables as aglycones, esterified glycosides or oligomers forms, with a phenylpropane unit present in their chain. They are classified into five subclasses: lignans, neolignans, norlignans, hybrid lignans and oligomeric lignans. These subclasses differ by the carbon chain structure, the way in which oxygen is incorporated into the structure and the cyclization pattern [87,98].

Tannins have high molecular weight, can either be water soluble or insoluble, and are able to combine with proteins or other polymers. There are two classes of tannins: condensed tannins and hydrolysable tannins. Both are chemically reactive, forming intra- and inter- hydrogen bonds, and precipitating molecules [89,99,100]. Moreover, condensed tannins (also known as proanthocyanidins) are found in superior plants and are formed by catechin polymers and leucoanthocyanidins [89,101]. Hydrolysable tannins are formed by a glucose or by a polyhydric alcohol skeleton, esterified with gallic or hexahydroxy diphenyl acids, and are prone to weakening the acid–base balance and interfering with some enzymatic hydrolysis reactions [100].

Phenolic compounds metabolism promptly occurs in the human organism and relies on physicochemical properties such as configuration, lipophilicity and solubility [96]. Within each of their structures, the absorption process happens when aglycones are absorbed in the small intestine and then metabolized before reaching systemic circulation. Hydrophilic flavonoid glycosides are transported across the small intestine by Na+-dependent cotransporters in the intestine. However, an alternative mechanism proposes that flavonoid glycosides must be converted into aglycones by lactase phlorizin hydrolase enzyme (LPH) in the small intestine brush border membrane. Thus, the non-degraded flavonoid glycosides by LPH are absorbed in the colon by means of the action of the microbiota which are able to rearrange theirs structures, adding or removing methoxy and hydroxyl groups, but in smaller amounts due to the absorption limitation of the site [102]. Regarding the absorption process of these compounds, the molecular weight is considered a key factor. While phenolic compounds with low molecular weight such as isoflavones, flavones and catechins are easily absorbed, proanthocyanidins, which have a high molecular weight, have more difficulty during this process [103].

#### Phenolic Compounds Quantification

The identification of specific phenolic groups in foods is difficult to assess, since they are susceptible to interference; therefore, the most common determination is given in total phenolic compounds [104]. To isolate compound structures, the choice of an adequate solvent and method of extraction is necessary, since many factors can affect this process. The exposure to heat can either enhance or diminish phenol extraction and antioxidant activity. While exposure to heat may facilitate the extraction of phenolic compounds due to plant cell disruption [105], excessive heat may decrease phenol and antioxidant activity [106]. Moreover, the time of extraction also interferes with phenol obtainment [107]. Singh et al. [104] considered the sample powder extraction the most recommended protocol among all methods, since it promotes a larger contact surface and the rupture of the sample’s cell wall, optimizing the process.

One of the most used methods to assess phenolic compounds is the Folin–Ciocalteu reagent (FCR) method. First, this method was used for protein analysis, taking advantage of the reagent’s activity toward protein tyrosine (containing a phenol group) residue [108]. Thereafter, the assay was extended to total phenols analysis in wine and, ever since, many applications for this method have been found [109].

A proposal for standardizing the routine quality control and the antioxidant capacity measurement of food products and dietary supplements with the FCR method was made [110]. The FCR method relies on the electron transfer in an alkaline medium from phenolic compounds to phosphomolybdic/phosphotungstic acid complexes to form blue complexes that are determined spectroscopically at approximately 760 nm [109,111]. The FCR method, also known as the total phenols (or phenolic) assay, assesses a sample’s reducing capacity without reflecting the name “total phenolic assay”. Diverse publications applied the total phenols assay by FCR and an ET-based antioxidant capacity assay and frequently found excellent linear correlations between the results of FCR and the antioxidant activity. This can be explained by the similarity of FCR and ET-based methods, both assessing a sample’s reducing capacity. However, the use of FCR along with an ET-based method is redundant [112].

## 3. First Issue: Common Antioxidant Activity Methods In Vitro

### 3.1. DPPH

The 1,1-diphenyl-2-picryl-hydrazyl (DPPH) is considered a stable free radical by virtue of the delocalization of the spare electron over the molecule, which prevents the molecules from dimerizing, as would happen with most other free radicals. This configuration confers a violet color, with the absorption band in an ethanol solution centered at about 520 nm. When a substance that could donate hydrogen is mixed with a DPPH solution, it produces the reduced form of DPPH with loss of the violet color, expressed by the Equation (1), where DPPH is represented by Z• and the donor molecule is represented by AH, resulting in the reduced form ZH, and A• is a free radical produced in the first step.

Z• + AH = ZH + A•(1)

Then, the free radical will undergo further reactions (Equation (2)), and create a stable product, represented by RS-SR [113]. While DPPH is able to accept an electron or hydrogen radical to become a stable, diamagnetic molecule, it can be oxidized only with difficulty, and then irreversibly [114].

RS• + RS• = RS − SR(2)

DPPH is a rapid, simple, inexpensive and widely used method [115] to assess antioxidant properties in wheat grain and bran, vegetables, conjugated linoleic acid, herbs, edible seed oils, and flours in several solvent systems including ethanol, aqueous acetone, methanol, aqueous ethanol and benzene [116,117]. It can be used in biological samples and in solid or liquid samples [115], even though its use is questioned in the evaluation of amino acids, since it was demonstrated to be insensitive to these compounds. Only cysteine (Cys) displayed noticeable DPPH• scavenging activity among the several protein amino acids tested [118].

Another issue is the validity for plasma samples. The alcohol medium precipitates the proteins within the plasma, which is not useful for these kinds of samples [115]. Although the method is simple, it is limited because DPPH interacts with other radicals. The time response curve to reach the steady state is not linear with different ratios of antioxidant/DPPH [119,120]. Due to the fact that several events can interfere in antioxidant assays, the DPPH free radical method is performed at room temperature and, thus, eliminates the risk of the thermal degradation of the molecules tested [121]. Moreover, for being a colorimetric assay, the method can be distributed to compounds that confer colors, such as carotenoids [122], and, therefore, the results should be adjusted to correct the access of antioxidant activity. Conversely, the DPPH assay can be influenced by the reaction time; some compounds react immediately with DPPH depending on their structure, while for a number of compounds already tested, the reactions might be slower, due to the presence of more complex mechanisms [7,119].

Once DPPH is applied to evaluate the overall antioxidant capacity, it allows to us assess weak antioxidants, which, in turn, ends up slowing down the reaction time [119]. Regarding the reaction time, the original method recommends 30 min for reading [123]. Nonetheless, the rate of reaction varies among substrates [119,121], and it has been proposed to follow the reaction to completion (plateau) [120,124,125]. Additionally, the reaction time can be influenced by the sample concentration and the nature of the antioxidants, as the fixed-time experiment underestimates radical scavenging activities through slow reacting molecules [126] and, therefore, achieving the plateau state could be more accurate for assessing the antioxidant capacity in these samples.

The efficient concentration value (EC50) is the quantity of antioxidants capable of decreasing the DDPH• concentration by 50% [97]. This parameter is used in the interpretation of the DPPH method results and was introduced by Brand–Williams and co-workers [113,119,121]. However, some issues have been reported on the use of the EC50 value. Since the determination made an analogy to lethal dose (LD50), one could be led to think that the assessment takes place in a biological system [113,124,126].

Another parameter used to evaluate is the TEC50, which is the time necessary to reach the steady-state EC50 concentration. The antiradical efficiency (AE) is derived from these parameters, represented by the following equation (Equation (3)):

AE = 1/EC50∙TEC50(3)

While EC50 is a common method, AE is less popular. However, some authors prefer to use AE, since the interpretation is clearer [127]. The higher value of AE is equal to a higher antioxidant content, in opposition to EC50. Aside from that, some authors have simplified the equation, not taking into account the time of reaction [127,128,129], which does not fulfill the purpose of the parameter to consider slow antioxidants, and instead seems to only simplify the interpretation of the method. Conversely, antiradical power (ARP) is the reverse of EC50, and, similarly to AE, the higher the ARP value, the more efficient the antioxidant activity is [119]. The use of this parameter can also facilitate the comprehension of antioxidant efficiency.

### 3.2. TEAC/ABTS

The 2,2′-azino-bis-3-ethylbenzthiazoline-6-sulphonic acid (ABTS) assay expressed in Trolox equivalent antioxidant capacity (TEAC) is a spectrophotometric assay, based on the extent of the ABTS•+ radical cation reduction in an aqueous medium at a fixed time point, rather than the rate of reduction [130]. Trolox is a synthetic antioxidant (water-soluble vitamin E analogue) used as a standard of comparison, giving rise to TEAC. The TEAC refers to the millimolar concentration of a Trolox solution with the antioxidant capacity equivalent to a 1 mM solution of the substance under investigation. Thus, TEAC reflects the relative ability of hydrogen or donating-electron antioxidants to scavenge ABTS radical cation in relation to Trolox [18].

ABTS presents a long-wavelength absorption spectrum with maxima at 660, 734 and 820 nm. Originally, this assay measured the ability of a compound to reduce the ABTS radical, formed by ABTS with a ferryl myoglobin radical species, generated by the activation of metmyoglobin with H_2_O_2_. However, the compound can also react with the ferryl myoglobin species, reducing them [131]. Aside from that, Miller and Rice–Evans (1997) found that the results of a myoglobin-ABTS assay and the direct reduction of the ABTS radical cation were very similar; therefore, the antioxidant activity was via ABTS scavenging rather than inhibiting the formation through the reduction of ferryl myoglobin or a reaction with H_2_O_2_. ABTS cannot be considered a pro-oxidant, since it is quite stable [132] when it occurs with DPPH. Although the ABTS assay is a well-known method to assess total antioxidant activity and is used in diverse samples, such as food and body fluids [133], some authors do not consider it an accurate method. Since it does not use a substrate to be oxidized, it cannot adequately mimic the processes in food and biological samples, meaning it is considered artificial [18]. Moreover, ABTS is not found in the human body, and thus it is recognized as a non-physiological assay [7]. ABTS shows a positive correlation with the DPPH method results and is strongly correlated with oxygen radical absorbance capacity (ORAC) from the USDA database when compared to the DPPH assay [134]. Floegel et al. [135] also found a better correlation between ABTS and ORAC database when analyzing fruits, vegetables and beverages. The authors suggested that ABTS better reflects the antioxidant content in different foods than DPPH. The correlation of results between the methods might be explained once all methods are associated with total phenolics content measured by the Folin–Ciocalteu method [135,136].

### 3.3. ORAC

The oxygen radical absorbance capacity (ORAC) assay uses β-phycoerythrin (β-PE) as an oxidizable protein substrate and 2,2′-azobis (2-amidinopropane) dihydrochloride (AAPH) as a peroxyl radical generator, or Cu21-H_2_O_2_ as a hydroxyl radical generator [131]. It consists of measuring the decrease in the fluorescence of the protein as a result of its conformation loss when it suffers oxidative damage, caused by the radicals in the test. The assay evaluates the ability of the antioxidants in the sample to protect the protein from oxidative damage [137,138].

The method using β-PE suffers from some disadvantages, such as β-PE presenting with great lot-to-lot variety, being photo-bleached under plate-reader conditions, interacting with some polyphenols via nonspecific protein binding, and losing its fluorescence even without a radical generator [131,135,137]. A modification of the method was proposed by Ou et al. in 2001, exchanging PE for fluorescein (3′,6′-dihydroxyspiro[isobenzofuran-1[3H], 9′[9H]-xanthene]-3-one) as a protein target, addressing the inconsistency problems regarding batches, photosensitivity and interaction with phenolic compounds owing to nonspecific protein binding, as occurred with PE [139]. Another variation of the method was described using pyrogallol red (PGR) as a probe, rather than fluorescein, in developing a food database in South America [140,141]. As seen when modifying the probe from β-PE to fluorescein, the results differed through the probe chance to PGR.

The ORAC method has been successfully used to evaluate antioxidant capacity in foods (tea, fruits, vegetables, herbal mixtures) [142,143] and in biological systems [144,145]. ORAC measurement has an advantage over other methods, since there is a database providing food equivalents in ORAC values, which might provide valuable information in assessing antioxidant intake [146] along with other databases containing ORAC values available for products from the South Andes and South America [147]. The ORAC database used to be available on the USDA site; however, in 2012, the database was withdrawn under the allegation that “the values indicating antioxidant capacity have no relevance to the effects of specific bioactive compounds, including polyphenols in humans”, but it is still available on a private site with updated information.

### 3.4. FRAP and TRAP Assays

The ferric ion (Fe^3+^) reducing antioxidant power (FRAP) and the total antioxidant radical scavenging parameter (TRAP) are tests that evaluate the total antioxidant capacity of a compound using the same prerogative; that is, the capacity of antioxidants to reduce a colorless complex to a color that can be analyzed by spectrophotometry with the proper pattern, average length and wavelength. However, FRAP is an electron transfer-based method, while TRAP is classified as a hydrogen atom transfer-based method; both can be used to assess the total antioxidant capacity of a compound or an organism (blood serum or macerated tissues) [10,148].

The FRAP assay chemical reaction is based on the capacity of a Fe^3+^ complex reduction. When a substance has antioxidant properties, the colorless complex is changed into a blue-colored one [Fe^2+^(TPTZ)_2_ ]^2+^ in acid medium, also known as Prussian blue. As a colorimetric assay, color changes can be measured at 593 nm on a spectrophotometer; the standard used is a ferrous ion solution, and Trolox (6-hydroxy-2,5,7,8-tetramethylchroman-2-carboxylic acid), a soluble analogue of vitamin E, is used as a positive control. The result is expressed in FRAP units, since the FRAP unit is related to the reduction of ferric ion 1 M into one ferrous ion [149].

The tripyridyltriazine (TPTZ) is used as the linking ligand of the iron ion but, more recently, potassium ferricyanide has been the most common ferric reagent used in the reaction. The acquisition of Prussian blue by the end of reaction can be performed in two different ways: Fe^3+^ can be reduced to Fe^2+^, which binds to ferricyanide to yield Prussian blue, or antioxidants can reduce ferricyanide to ferrocyanide, which binds the free Fe^3+^, and also results in Prussian blue [150].

There are some limitations of the FRAP assay, such as the redox potential of the Fe^2+^/Fe^3+^ pair that can induce a false Fe^3+^ reduction when testing compounds with a lower redox potential, the rapid time of reaction which considers that redox reactions are fast, but is not always the case, and the hydrophilic environment of the reaction preventing the hydrophobic compounds from being efficiently tested [149].

For the hydrophilic/lipophilic limitation, Berker, Demirata and Apak [122] proposed the use of a 1:9 (*v*/*v*) water-acetone medium in the reaction in the absence of the methylated β-cyclodextrine (RMCD) solubility potentiator to assess the antioxidant capacity of both hydrophilic and lipophilic compounds simultaneously. Their results were similar to that using the solubility potentiator, which can support a more affordable experiment, not losing its reliability. Wojtunik-Kulesza [149] tested the addition of a surfactant (Tween-20) to enhance some monoterpenes’ solubility and observed a reduced clouding in the studied solutions and a false positive effect, and also applied thin-layer chromatography (TLC) with FRAP, demonstrating that the combined methods could be used as a fast, more reliable and reproducible way to determine the reducing activity of extracts [122,149].

Another derivation of the FRAP method was performed by Berker et al. [151] to ensure a more optimized, reproducible assay and more linear results. For this purpose, they used ferricyanide and Fe (III) simultaneously to promote a more favorable redox environment for a wider range of antioxidants. To avoid the precipitation of Prussian blue, the researchers added sodium dodecyl sulphate (SDS), a tensoactive compound, and adjusted the optimal pH to 1.7, maintaining the redox activity of ferric ion while preventing its hydrolysis.

The FRAP assay is a method widely used to assess total antioxidant capacity of foods, beverages, plant extracts, essential oils and biological fluids, and can be also used as a prognostic biomarker for several conditions, such as hypertension, acute myocardial infarction followed by cardiogenic shock and chronic kidney disease [150,151,152,153,154].

The TRAP method was developed by Wayner et al. [155] and, originally, it was based on measurements of induction times in the oxidation of lipids, measuring the total peroxyl radical-trapping ability of plasma. The plasma peroxidation was triggered by ABAP [2,2′-azo-bis- (2-amidipropropane hydrochloride)] thermolysis to yield peroxyl-radicals, and the time of oxygen uptake inhibition by plasma antioxidants was registered. The inhibition time of O_2_ absorption is called the induction period, and is quantitively measured as the TRAP index (number of mols of ROO^•^/liter of fluid). In this way, TRAP can be calculated as follows: TRAP = R _ROO_ x τ_plasma_, where R_ROO_ is the free radical formation rate and τ_plasma_ is the delay time in the oxygen consumption.

Considering the information obtained from this review, some modifications were made to the original method as follows: (i) the incorporation of a chemiluminescence enhancer such as luminol proposed by Lissi et al. [156], where luminol could oxidate from ABAP-derived ROS, losing its hydrogen atom and then emitting fluorescence; (ii) the total antioxidant parameter (TAR), which represents the total capacity of all antioxidants present in the sample to modulate the damage associated with enhanced free radical production; (iii) and the validated adaptation by Dresch et al. [157] that proposes the use of the area under the curve (AUC) to evaluate homogenously different kinds of antioxidants and samples, including those without presenting a lag phase and/or samples presenting multiple antioxidant curve profiles. Instead of measuring a steady point of the curve, the assessment of the AUC combines the lag time-based method, the initial rate-based method and samples with complex kinetics.

The TRAP assay measures the antioxidant/compound’s capacity to scavenge luminol-derived radicals generated from ABAP or 2,20-azobis(2-amidinopropane) dihydrochloride (AAPH) thermolysis, which is the most used peroxyl radical generator in hydrophilic systems, using β-phycoerythrin, fluorescein, dihydrofluorescein diacetate (DCFH-DA) or luminol as fluorescent probes and free radical sources as well. The reactions are usually performed at 21 °C in an alkaline medium (pH 8.6), sodium phosphate or glycine buffer, and the chemiluminescence is captured by a luminometer or a scintillation counter [150].

Some practical uses of the TRAP assay are its correlation with pathological conditions, which could be used as an oxidative stress biomarker for diagnostic, prognostic and epidemiological purposes. Praud et al. [158] found an inverse relation between TEAC, FRAP and TRAP with the gastric cancer odds ratio, and concluded that an antioxidant capacity-rich diet (vegetables, fruits, whole cereals and moderate consumption of wine) was able to reduce gastric cancer risk.

Siegfried and Shui [159] observed that patients who had undergone a vitrectomy had significantly lower TRAP results, i.e., decreased antioxidant capacity of aqueous humor following vitrectomy in a prospective cross-sectional study (*n* = 288 eyes). The authors concluded that an elevated intraocular oxygen pressure with antioxidant depletion following the vitrectomy procedure may suggest an alteration of the intra-ocular oxidant–antioxidant balance, and the oxidative stress generated from that imbalance may contribute to intraocular pressure elevation and an increased risk of glaucoma.

### 3.5. DPPP

Okimotoa et al. [160] tested the ability of diphenyl-1-pyrenylphosphine (DPPP) to be used as a fluorescent probe to monitor lipid peroxidation in cells. The fluorescence of DPPP-labeled cells was measured with fluorescence microscopy and with a spectrofluorometer for cell-free systems. Subsequently, the flow cytometer was also used as a means of fluorescence detection, and DPPP proved to be an excellent probe for long-term cell oxidation studies [161].

Adaptations of the assay based on the oxidation of a non-fluorescent DPPP to a fluorescent phosphine oxide were developed in order to quantify the activity of 15-lipooxygenase (15-LOX) and discover its inhibitors. This enzyme plays important roles in humans, being part of various conditions related to inflammatory processes (diabetes, obesity, cardiovascular diseases among others) but also related to homeostasis and physiological events [162,163,164]. The assay principle is based on 15-LOX, which catalyzes the introduction of molecular oxygen into polyunsaturated fatty acids, such as arachidonic and linoleic acids. The enzymatic reaction gives rise to lipid hydroperoxides that play the role of oxidative mediators due to their instability, leading to more free radicals and oxidation reactions with DPPP, resulting in a fluorescent phosphine oxide. In this way, DPPP can be used both as a 15-LOX marker and as an identification tool for 15-LOX inhibitors, or as a method to study lipid hydroperoxides in various samples [164,165].

The final fluorescent compound of the reaction is not sensitive to temperature and proved to be stable for more than 2 h after the experiment. It can be performed in enzyme (biochemical) or cell-based assays using a black 96- or 384-well plate, being amenable for high-throughput screening and scaling down. Another feature of the DPPP assay is the possibility to determine the potency of the 15-LOX or any other treatment inhibition on the oxidation process by analyzing IC50 values, and it is a peroxidase-free assay not relying on a secondary enzymatic step. The fluorescence signal intensity can be measured with a fluorescence plate reader from the excitation wavelength of 363 nm and the emission wavelength of 380 nm [163].

Several reactions in solutions are initiated by factors such as heat, light, enzymes, metals and metalloproteins that trigger lipid oxidation, generating lipid hydroperoxides that can also trigger other peroxidation chain reactions. These compounds do not add any odor or flavor to food products, but they may decrease the nutritional value. Proteins can also be targets of free-radicals and form hydroperoxides as well, thus contributing to carbonylation and cross-linkage formation. When it comes to protein oxidation and protein hydroperoxide formation, the food industry faces a more complex scenario once these reactions lead to protein aggregation and loss of solubility, interfering in the sensory quality and in the nutritional value which could affect the product shelf-life [165].

Adaptations of the original DPPP method allowed the determination of lipid and protein hydroperoxides at the same time, which appears to be an important tool for food quality control. The method combines a well-known lipid extraction step [166], which can be directly performed in chloroform and water/methanol phases followed by centrifugation and the acquisition of three distinct phases: an upper water/methanol layer corresponding to water-soluble secondary lipid oxidation products, a bottom chloroform layer corresponding to lipid hydroperoxides, and a middle thin layer, also called a disc, corresponding to protein hydroperoxides and precipitated proteins. In this way, it is possible to isolate each layer and perform the DPPP assay for lipo- and water-soluble hydroperoxides with a single sample. The adapted method was considered rapid, with high accuracy, simple and reproducible, being useful in the analysis of lean fish or meat as well as protein hydrolysates. This assay can be performed either using a 96-well plate and a spectrofluorometer or using the macerated sample on a fluorescence microscopy which allows not only lipid and protein hydroperoxide quantification, but also the visualization by fluorescence imaging as a complimentary analysis or in the absence of a spectrofluorometer. The fluorescent signal of DPPP is not affected by pigments absorbing at 560 nm or by iron and chelators present in muscle meat [165,167].

The main advantages and disadvantages of the discussed methods are shown in Table 1.

Different medicinal plants have been used as a source of natural antioxidants, phytochemical properties and antioxidant capacity. Yousfi et al. [86] evaluated the phenolic composition and antioxidant activity of hydroethanolic extracts from the leaves of *Salvia officinalis*, *Rosmarinus officinalis*, *Olea europaea*, and *Punica granatum*, as well as the leaves and stems of *Ruta graveolens*, *Mentha piperita* and *Petroselinum crispum*. According to their results, P. granatum has the highest content of antioxidant compounds such as total phenols, flavonoids, tannins and ortho-diphenol and the highest antioxidant capacity, measured by the DPPH, FRAP and ORAC methods. Furthermore, a strong correlation between the phenolic composition and the antioxidant properties of plants was observed by the authors [86].

Mwamatope et al. [84], when evaluating the methanol extract of plants of the species *Senna singueana*, *Melia azedarach*, *Moringa oleifera* and *Lannea discolor*, from the northern Malawi, Africa, also demonstrated a direct positive relationship between the content of total phenols and the antioxidant capacity, and the highest total phenolic content (27.64 ± 0.09 mg GAE g DW) was quantified in the bark of *L. discolor*, a species belonging to the Anacardiaceae family that has been demonstrated as a good source of antioxidant natural and used in the treatment of the effects of cancer. Other plant species also have antioxidant potential. In fact, the antioxidant activity using DPPH, reducing power and metal chelating assays was determined in crude juices extracted by the hydraulic pressing of leaves of *Ficus carica* (fig), *Psidium guajava* (guava), *Olea europaea* (olive) and *Punica granatum* (pomegranate) leaves and bark, all plants from Giza, Egypt. According to the results, the raw juice from F. carica bark had the highest antioxidant activity compared to the juice from other medicinal plants and from the *F. carica* leaves, while the flavonoid content was similar in all species studied [81].

## 4. Second Issue: Evaluation of Antioxidant Activity in Biological Systems—The Role of Enzymes in Redox Cellular Signaling

Cells have complex antioxidant systems to regulate cellular redox and to protect them against free radical damage. This control happens with enzymatic and non-enzymatic systems. Non-enzymatic antioxidants rely on the action of glutathione, melatonin, vitamins C and E, carotenoids and flavonoids, while enzymatic systems rely on several enzymes, including superoxide dismutase (SOD), catalase (CAT), glutathione peroxidase (GPx), peroxiredoxin and glutathione S-transferase (GST). Nicotinamide adenine dinucleotide phosphate (NADPH), reduced glutathione (GSH) and thioredoxins act with these enzymes in defense against the damage caused by free radicals [168].

**Figure 1:**
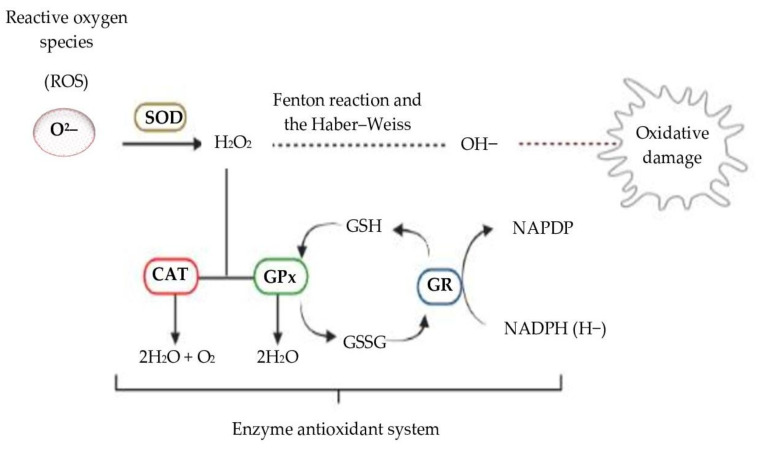
Integration of enzymes that participate in the antioxidant defense of the enzymatic antioxidant system. Note: During the processes of aerobic metabolism, reactive oxygen species are formed, such as superoxide (O²−) radicals. The enzymatic system is the first to act to prevent the accumulation of these molecules and their intermediates. Superoxide dismutase (SOD) catalyzes the dismutation of the superoxide radical into hydrogen peroxide (H_2_O_2_), which despite not being a free radical, due to the absence of unpaired electrons in the last layer, H_2_O_2_ is an oxygen metabolite that participates in the Fenton and Haber–Weiss reaction and results in hydrogen peroxide (OH−)production, responsible for major cellular damage. To avoid this, other enzymes act in conjunction with SOD, namely catalase (CAT) and glutation peroxidase (GPx). CAT catalyzes the reduction of H_2_O_2_ to water (H_2_O) and oxygen (O_2_). GPx catalyzes the reduction of H_2_O_2_ to H_2_O at the cost of converting reduced glutathione (GSH) to glutathione disulfide (GSSG). In the oxidation process, glutathione reductase (GR) regenerate GSH in the presence of NADPH (H+) as a cofactor. The integrated action of these enzymes is responsible for protecting cells from oxidative damage.

The SOD, CAT and GPx enzymes that make up the enzymatic defense system work together to control the formation of free radicals, thus protecting cells from oxidative damage as shown in Figure 1 below:

### 4.1. Reduced Glutathione

Reduced glutathione (GSH) is a compound with low molecular weight composed of glycine and cysteine glutamic acid. GSH is present in all plant and animal cells, and acts as an intra-cellular reductant that protects cells against free-radicals, peroxides and other toxic compounds [169,170]. Besides the antioxidant action, GSH has also different functions not related to defense against free radicals and participates in the detoxification process of electrophilic compounds (xenobiotics), the metabolization of prostaglandins and leukotrienes, the transport of amino acids and the absorption of micronutrients from the intestine, such as iron and selenium. Nevertheless, the predominant role of GSH is as an antioxidant [169,171].

It is proposed that, in the process of regenerating other antioxidants, GSH is oxidized to glutathione disulfide (GSSG), which can be reduced by glutathione reductase (GR) to regenerate GSH in the presence of NADPH (H+) as a cofactor [172]. However, the mere redox state of GSH/GSSG was questioned by Flohé (2013), who concluded that GSH/GSSG balance does not exist in biological systems [173]. Despite this, the same author affirmed that the thiol/disulfide ratio might still reflect what happens in the organism and has diagnostic/prognostic value.

GSH ameliorates free radical damage during enzymatic and non-enzymatic reactions. It regenerates other oxidized small molecule antioxidants, such as vitamin C and vitamin E [174], and is involved in the repair of protein molecules, nucleic acids and lipids damaged by peroxidation, and in the maintenance of sulphydryl protein groups in the reduced state [175,176,177]. Intracellular glutathione in its reduced form (GSH) has a significant role in maintaining the redox homeostasis, since it can act as a direct antioxidant and as a substrate for GPx, the radical scavenging enzyme [178]. The GSH/GSSG ratio is used to evaluate the intracellular oxidative stress in which an increased ratio of GSSG/GSH indicates increased oxidative stress [173,179]. The quantification of GSH and GSSG can be determined enzymatically using the Brehe and Burch method [180]; another way to quantify GSH is using the fluorometric method, also described by Brehe and Burch [180], using a smaller amount of tissue.

### 4.2. Glutathione Peroxidase

Glutathione peroxidase (GPx) is the general name of an enzyme family with peroxidase activity whose main activity is to protect the organism from oxidative damage. GPx reduces lipid hydroperoxides to their corresponding alcohols and reduces H_2_O_2_ into water [181]. GPx 1–4 contain a selenocysteine (SeCys) at the active site, while GPx 5, 7 and 8 have a Cys instead of SeCys at the active site; therefore, they are called non-selenium GPx. GPx 6 has a SeCys at the active site in the human organism, and other organisms contain a Cys at the active site [182,183]; the SeCys-containing GPx has a greater ability to reduce H_2_O_2_ than the Cys GPx [182].

Regarding the SeCys GPx, the selenocysteine in the active site is responsible for the catalytic activity. It was first isolated and described by Flohé and co-workers [183] in 1973, when it was confirmed that the enzyme contained selenium atoms. Furthermore, selenium is considered an essential element by the World Health Organization (WHO), regarding its antioxidant properties and, therefore, the recommended dietary allowance (RDA) and the estimated average requirement (EAR) for this element is based upon the maximization of seric GPx [184].

GPx, along with SOD and CAT, are the most important antioxidant enzymes and are usually used as biomarkers to indicate the production of ROS [185,186]. The activity of GPx can be accessed with a kinetic method first described by Strauss et al. [187], which involves two steps: (i) the neutralization of H_2_O_2_ by GPx in the presence of GSH and (ii) recycling of the resulting GSSG by GR in the presence of NADPH; the consumption of NADPH is then used as a proxy for GPx activity.

### 4.3. Superoxide Dismutase

Metal ion-dependent superoxide dismutases (SOD) are essential components in the defense of the antioxidant system. There are three types of SODs present in mammalian physiology with tightly regulated localization patterns. That is, there are two copper/zinc containing members, CuZnSOD (SOD1), located within the cytosol, mitochondrial intermembrane space and nucleus, and EcSOD (SOD3), being the predominant antioxidant enzyme, secreted into the extracellular space [188,189,190]. Moreover, manganese-containing SOD (SOD2) is located in the mitochondrial matrix and shows minimal similarities with other SODs [189].

SOD is an enzyme that acts to regulate superoxide levels through the conversion of superoxide to H_2_O_2_ and cellular oxygen. The antioxidant function performed by SOD has been the subject of several studies due to its potential as a therapeutic agent in the treatment of inflammatory disorders [191,192,193,194].

Topical administration containing the SOD enzyme in patients with atopic dermatitis demonstrated the ability to significantly reduce the production of nitrogen oxide (NO) and the secretion of inflammatory cytokines (TNP-a, IL-1, IL-6, etc.) responsible for pathogenesis of the disease [195]. In addition to the known action of SOD in diseases such as cancer, diabetes, bronchopulmonary dysplasia and arthritis, there are currently records of the action of SOD mimetics as a therapeutic against cancer progression [196]. The action of SOD mimetics is also confirmed as protecting the epithelial tissue of the prostate and inhibiting the development of radiation-induced cancer [197], by increasing the concentration of H_2_O_2_ in the tumor [198]. The existence of another benefit is the improvement of liver function due to improved insulin resistance and reduced inflammation promoted by type 2 diabetes [199].

Radiation-induced free radical production directly results in the development of fibrosis, causing damage to normal tissues. Thus, SOD overexpression attenuates the induced damage, and the addition of SOD mimetic after total radiation exposure demonstrated protection against pulmonary fibrosis [200]. Another model of pelvic irradiation concluded that there is prevention of chronic and acute damage due to SOD mimetics by myofibroblast differentiation [201]. In contrast, the reduction in the activity or mutation of this enzyme is associated with the genesis of several diseases, including degenerative, renal, ocular and auditory diseases [202,203].

All SODs require metal cofactors to catalyze one-electron oxidation, followed by one-electron reduction of two O_2_•– anions to affect disproportionation. Therefore, changes in the levels of these cofactors, especially the presence of manganese (Mn), zinc (Zn) and copper (Cu) found in the SOD structure, can reduce the efficiency of these enzymes [203]. Considering their distinct localizations and the membrane impermeability for O_2_•–, each member of the SOD family is expected to have specific compartmentalized roles for the regulation of redox-sensitive transcription factors, mitochondrial oxygen level sensing and the protection of surrounding tissue from oxidative inflammation during infections [204,205,206,207].

Thus, SOD1 is responsible for destroying free radicals in the body and plays an important role in apoptosis signaling and oxidative stress [208], while EcSOD is a superoxide scavenger, which is a precursor to diverse ROS and NOS [209]; however, its redox modulation effect is not limited to controlling the levels of this radical. EcSOD suppresses the accumulation of superoxides, preventing spontaneous dismutation of superoxides into H_2_O_2_. Beyond that, generation of •OH via the Fenton reaction and the Haber–Weiss reaction might be prevented by EcSOD, which, in turn, might be responsible for preventing the superoxide-mediated oxidation of NO•, controlling the formation of ONOO– [210]. SOD assays use the competitive kinetics of O_2_•– reduction of cytochrome c (probe) and an O_2_•– scavenger (sample) [111]. The method has been adapted to a microplate format [211]. Since cytochrome c can be reduced directly by antioxidants, it can also inhibit the xanthine oxidase, which makes this method unsuitable for quantifying non-enzymatic antioxidants [212].

### 4.4. Catalase

Catalase (CAT) is an enzyme found in nearly all living organisms, and it is part of the first line of antioxidant defense, preventing the formation of free radicals. Similarly to GPx, CAT also reduces H_2_O_2_ to H_2_O and molecular oxygen completes the detoxification process imitated by SOD [191,213,214,215]. Moreover, CAT reacts with hydrogen donors (such as methanol, ethanol, formic acid, phenols with peroxidase activity). CAT activity takes place in two steps: (i) a molecule of H_2_O_2_ oxidizes the heme group to an oxyferryl species (a porphyrin cation radical is generated when one oxidation equivalent is removed from iron and one from the porphyrin ring), and (ii) a second H_2_O_2_ molecule acts as a reducing agent to regenerate the resting state enzyme, with an oxygen molecule and H_2_O as the final products [213].

CAT enzymes are abundant in cells, where they continually scout for H_2_O_2_ molecules and, being extremely efficient, are able to break down millions of H_2_O_2_ molecules in one second [216]. Animals use catalase in organs, with particularly high concentrations occurring in the liver [217]. The deficiency or mutation of CAT is linked to various disease conditions and abnormalities [218]. CAT is quantifiable, determined by spectrophotometry, by following the action of catalase with H_2_O_2_ based upon the measurement of ultraviolet absorption of peroxide. The breakdown of H_2_O_2_ follows first order kinetics under a variety of conditions and increases linearly with CAT concentration. Although CAT is measured by spectrophotometry, the interference of other compounds is minimized by using a wavelength at which the absorption of the foreign components is at a minimum, in the region from 200 to 300 nm, or by preparation of a new calibration curve to which an appropriate amount of the absorbed foreign substances has been added [219].

### 4.5. Thiobarbituric Acid-Reactive Substances

Thiobarbituric acid-reactive substances (TBARS) are used as markers of lipid peroxidation and products that reflect oxidative damage to DNA [17]. The assessment occurs by spectrophotometry, which measures malondialdehyde (MDA) and other aldehydes produced by lipid peroxidation induced by hydroxyl free radicals [220]. The validity of this assay is based on the facility of polyunsaturated fatty acids being targets for oxidants and, because the process of lipid peroxidation is initiated as a self-sustaining free radical chain process, the accumulation of peroxidation products provides the most common biochemical marker of oxidative stress [221]. Many criticisms have been made about the use of the TBARS method to assess oxidative processes in complex biological systems. The method is a colorimetric assay in which the formation of MDA and products leads to a pink color without interference in vitro. Concerning more complex systems, many compounds (including simple and complex carbohydrates, protein oxidation products, and nucleic acid oxidation products) react with thiobarbituric acid to produce colored adducts. Thus, the use of TBARS as a single indicator of lipid peroxidation in complex biological systems is not appropriate [222]. This method could be used in combination with other techniques, such as separation and mass spectroscopic analysis of thiobarbituric acid products, particularly MDA, and has value in accessing the role of lipid peroxidation in oxidative stress, as proposed by Kadiiska et al. [223].

## 5. Third Issue: Bioavailability of Natural Antioxidants and the Role of Their Antioxidant Activity in Biological Systems

The bioavailability of antioxidants varies greatly, and the most ingested compounds are not necessarily those with major concentrations of active metabolites in the target tissues [224]. The bioavailability can be affected by several factors, such as food source and chemical interactions with other compounds [225]. Antioxidants present in foods are usually found with other macromolecules such as carbohydrates, lipids and proteins in the food matrix, while in plant tissues, the most predominant form of compounds found are carbohydrates, especially in free conjugated forms [226].

The results of the studies on polyphenols, vitamins and carotenoids are summarized in Table 2, which describes the tested compound, study design, results on bioavailability (if analyzed), tests performed, acquired results and related antioxidant activity as a brief result of each study analyzed in the sections below.

The studies on polyphenols’, vitamins’ and carotenoids’ bioavailability and action using animal models or clinical trials in the following sections are summarized in Table 2

### 5.1. Polyphenols

The absorption of polyphenols from the diet was first thought to be minimal, since the majority of food flavonoids are bound to glycosides [94], and it was supposed that only aglycones could cross freely into the bloodstream from the gut wall because there is no specific enzyme in the gut that could transport or break glycosidic bonds [224]. In order to enable flavonoid absorption, glycosides must be removed from the flavonoid skeleton [227], which is performed by the action of enzymes present in the small intestine [228]. Nonetheless, not all flavonoids are readily absorbed in the small intestine, passing into the large intestine, where they can be degraded by the colonic microbiota into simple phenolic acids, and exert antioxidant activity in the intestine environment or absorbed into the bloodstream [229].

It is possible to quantify flavonoid-derived secondary metabolites in the blood and urine after the ingestion of foods containing flavonoids; however, unconjugated flavonoids in the original form can be found in small amounts in the same sample [226]. This fact suggests that the flavonoid secondary metabolites enter into the circulation and may exert biological effects in the body [228,230].

The major phenolic compounds in the human diet are the phenolic acids. These compounds are classified into two classes: cinnamic acid derivatives and benzoic acid derivatives [231]. Phenolic acids are rapidly absorbed from 1–2 h after the intake of fruits and vegetables [232], since they are usually absorbed in the upper part of the gastrointestinal tract. Diverse polyphenols, such as caffeic, galic, coumaric, ferulic and chlorogenic acids, can be actively absorbed in the stomach [231,233]. On the other hand, the small intestine also is another absorptive site, although the absorption of aglycone phenolic acids occurs to different degrees. Generally, esterified phenolic acids have a lower bioavailability, with only 0.3–0.4% of original intake, and need to be hydrolyzed in the enterocytes to reach the bloodstream, which does not occur in an efficient way in the ester bonds [234].

The bioavailability of guaraná (Paullinia cupana) catechins was assessed by Yonekura et al. in a clinical trial [235]. The participants (healthy overweight adults, *n* = 12) were evaluated for 15 consecutive days, followed by intervention, with a daily intake of powdered guarana seed (3 g) containing 90 mg (+)-catechin and 60 mg (−)-epicatechin. Blood was collected on the first and last day from fasting individuals and 1 h after guarana intake, and the bioavailability of catechins was assessed. Plasmatic catechins, epicatechins and their metabolites were found 1 h after intake, and plasma ORAC was higher than before guarana intake, but it was not cumulative. Additionally, SOD, GPx and CAT methods were performed to assess antioxidant activity. After the trial period, GPx and CAT activity was augmented and remained higher even after plasma catechin clearance, while SOD remained the same. These findings show the bioavailability of catechins derived from guarana seed 1 h after intake, the direct antioxidant action due to increased levels of plasmatic ORAC and other assays performed (ex vivo LDL oxidation and H_2_O_2_-induced DNA damage in lymphocytes followed by the lymphocyte single cell gel electrophoresis), and the maintenance of SOD and CAT up-regulation.

The effects of green and black tea on oxidative stress were assessed in rats by Yao et al., where the animals were distributed over three groups: tap water (control), green tea (GT) and black tea (BT), both containing gallic acid, epigallocatechin, epigallocatechin gallate, epicatechin, and epicatechin gallate. The phenol constituents were determined by high-performance liquid chromatography (HPLC). During the experiment (5 weeks), the animals only had tea as a liquid source, and the consumption of food and water or tea was ad libitum. GSH was assessed in all groups and GT and BT showed increased levels compared to the control group in both the liver and lungs, and the GSH/GSSG ratio was higher as well. Lipid peroxidation was evaluated by TBARS, and the results did not differ in the groups [236].

Caffeic acid (CA) and ferulic acid (FA) were determined via gas chromatography mass spectroscopy (GC-MS) in individuals (*n* = 20) 1 h after the acute consumption of 400 mL of coffee or in the control group in a clinical trial developed by Lara-Guzmán et al. Neither CA nor FA were detected in the control group, while their presence was detected in the test group. ORAC, ferric reducing antioxidant power (FRAP) and trapping antioxidant potential (TRAP) were used to evaluate the antioxidant potential of these phenols in plasma samples. A significant increase in antioxidant activity was observed using FRAP and ORAC assays, while no difference was noted using the TRAP assay. The ex vivo LDL-oxidation was also performed and showed a highly significant delay of the oxidation reaction, once more demonstrating direct antioxidant activity and bioavailability of CA and FA from coffee [237].

The use of *Zingiber officinale* (ginger) in herbal medicine has been associated with the treatment of different diseases, including the prevention and treatment of cancer due to the presence of bioactive compounds with high anti-inflammatory, antimicrobial and antioxidant potential [238]. According to the observations of Ghafoor et al. [83], who evaluated the total phenolic content, total carotenoids, individual phenolic compounds and antioxidant activity by DPPH in dried ginger samples using different drying methods such as oven, microwave, freeze and room temperature drying, there is a significant difference in the drying processes and resulting bioactive compounds and antioxidant activity. In this case, the freeze-dried ginger rhizomes had a higher content of total phenolics (931.94 mgGAE/100 g), total carotenoids (13.17 μg/g) and antioxidant activity (82%) when compared to those samples dried using other techniques, presenting a good correlation between the total phenolic content and the antioxidant activity. Yousfi et al. [86] observed that the antioxidant capacity of ginger varies as a function of the type of preparation of the extract, such as in ethyl acetate, ethanol and water, as well as a function of the preparation temperature. In addition, the ethanol extract presented the highest polyphenol content at 20 and 40 °C (297.63 and 322.11 μg EAG/mg Ex) and higher antioxidant capacity compared to other extracts.

### 5.2. Vitamins C and E

Vitamins C and E have a more elucidated bioavailability than polyphenols. Vitamin C in foods is fairly available and easily transported in the intestine, since it is soluble in water, but is not stored in the body [239,240]. About 80–90% of the ascorbic acid is absorbed when the intake is up to 100 mg/day, decreasing rapidly with higher consumptions (500 mg/day) [240]. Vitamin E absorption depends largely on various factors, and one of the most important factors which affects bioavailability is the food matrix. The accessibility of α- and γ- tocopherols in different food matrices ranged from 0.5 in apples to nearly 100% in bananas and lettuce [47]. Besides that, lipids might enhance vitamin E bioavailability by providing a hydrophobic phase that could solubilize vitamin E. Moreover, the presence of lipids stimulates biliary secretion, resulting in micelle formation [241].

Iwata et al. [242] evaluated the effects of ascorbic acid supplementation in diabetic rats (streptozotocin-induced type 1 diabetes) after cerebral ischemia-reperfusion. Either 100 mg/kg of ascorbic acid or 100 mg/kg of water was administered through gavage for 2 weeks before the induction of ischemia as a pre-treatment. The diabetic state aggravated middle cerebral artery occlusion and reperfusion, causing more edema and enhanced infarct volume in brain tissue, but pre-treated animals with ascorbic acid significantly suppressed neuronal damage from nondiabetic and diabetic rats. In addition, the vitamin C pre-treatment suppressed the superoxide radical production, the caspase-3 activation and the inflammatory cytokines TNF-α (tumor necrosis factor α) and IL-1β (interleukin 1 β) in the peripheral ischemic area. It was possible to determine the upregulation of the sodium-dependent vitamin C transporter (SVCT2) on neurons and capillary endothelial cells and the presence of ascorbic acid in all nondiabetic cerebral tissue by immunohistochemistry protocol, but this response was suppressed in diabetic tissue. Thus, Iwata and colleagues concluded that vitamin C supplementation before brain ischemia and reperfusion acts to protect brain tissue from injury by means of an antiapoptotic and anti-inflammatory mechanism [242].

The effects of vitamins C (100 mg/kg/d) and E (100 mg/kg/d), β-carotene (15 mg/kg/d) and sodium selenite (0.2 mg/kg/d) combined and previously administered via gavage for 3 days on D-galactosamine-induced acute lung injury performed in rats were investigated by Bayrak et al. [243]. Four groups were described: control (saline), D-galactosamine (D-GaIN); D-GaIN + antioxidants and antioxidants. CAT, SOD, GSH and GPx activities were evaluated to assess antioxidant activity and MDA was conducted as well to assess lipid peroxidation. The tests were performed in lung tissue. CAT and SOD levels decreased as follows: D-GaIN > antioxidants > saline > D-GaIN + antioxidants. GSH levels were found as follows: D-GaIN + antioxidants > saline > D-GaIN > antioxidants. In the meantime, GPx levels decreased in the following order: D-GaIN + antioxidants > antioxidants > saline > D-GaIN. Lipid peroxidation increased in the following order: D-GaIN + antioxidants < saline < antioxidants < D-GaIN. Histologically, D-GaIN group presented with extensive edema, inflammatory infiltrate around the venules and honeycomb-like injury in the alveolar area, while the antioxidants-treated group had improved pulmonary edema improved but showed no effect in preventing pulmonary inflammation. The authors concluded that oxidative stress drives this experimental model and the administration of combined antioxidants induced antioxidant enzyme activities in the injured tissue, improving edema but being unable to prevent pulmonary inflammation [147].

Catal et al. [244] used the same injury model using D-GaIN to test the effects of antioxidant supplementation (100 mg/kg/day ascorbic acid +100 mg/kg/day α-tocopherol + 15 mg/kg/day β-carotene + 0.2 mg/kg/day sodium selenite) in the liver. The study described four groups: control, control + antioxidants, D-GaIN and D-GaIN + antioxidants. GSH and CAT activity were measured in the blood, with the higher GSH concentration in the control group, followed by D-GaIN + antioxidants, control + antioxidants and finally the D-GaIN group. CAT in blood decreased in the order: D-GaIN + antioxidants, control + antioxidants, control and D-GaIN group. Meanwhile, CAT, SOD, GST and GPx activities were evaluated in the liver tissue, and MDA was detected to assess lipid peroxidation. Both SOD and GPx were more concentrated in D-GaIN + antioxidants, followed by control, D-GaIN and control + antioxidants. GST in the liver tissue decreased in the following order: control, control + antioxidants, D-GaIN + antioxidants and D-GaIN; while CAT was higher in D-GaIN + antioxidants followed by control + antioxidants, control and D-GaIN. Lipid peroxidation increased in the following order: control, control + antioxidants, D-GaIN + antioxidants and D-GaIN. The D-GaIN animal model promoted an acute injury in the liver with edema, necrosis and mononuclear cell infiltrate, but the administration of antioxidants could enhance biochemical parameters and avoid severe histological damage, e.g., necrosis and edema, in addition to suppressing some mononuclear infiltrate [237,238]. It is possible to conclude that, when it comes to an acute liver injury animal model, the use of vitamin C, E, β-carotene and selenite combined presented antioxidant and anti-inflammatory properties by modulating the plasma and hepatic levels of enzymes and preventing/suppressing inflammatory features in the tissue.

### 5.3. Carotenoids

The bioavailability of carotenoids is related to absorption, which depends on the release of carotenoids from the food matrix, diffusion in lipid emulsion, solubilization in bile salts and pancreatic lipases and the formation of micelles to lead to transportation across the microvilli. Then, carotenoids may be uptaken by intestinal mucosal cells and incorporated in chylomicrons to finally reach the lymphatic system and circulation [245].

Several studies have been published focusing on carotenoid bioavailability [246,247,248,249]. It is clear now that carotenoid absorption is highly influenced by the food matrix and its extraction from it. The alteration of the food matrix can enhance bioaccessibility, augmenting carotenoid bioavailability, e.g., the lutein in chopped spinach is higher than in whole-leaf spinach [250]. Thermal or non-thermal treatment also increases carotenoid bioaccessibility [251,252].

The antioxidant and carotenoid bioavailability from microalgal biomass in a rat model was evaluated by Rao et al. [253]. Biomass containing a 200 µM equivalent of β-carotene, astaxanthin and lutein, derived from *Spirulina platensis*, *Haematococcus pluvialis* and *Botryococcus braunii*, respectively, was dispersed in olive oil and administered to rats for 15 days. The levels of carotenoids in plasma, liver and eye were examined by HPLC and confirmed with mass spectroscopy. CAT, SOD and peroxidase were assessed to determine the antioxidant activity and TBARS to assess lipid peroxidation. The carotenoids’ accumulation was higher in the liver, followed by the eye and then in the plasma. The activity of all antioxidant enzymes was enhanced after the administration of carotenoids and anti-lipid peroxidation activity (TBARS) was higher in the groups supplemented with carotenoids than in the control group. Astaxanthin from *H. pluvialis* showed better bioavailability and accumulation than other groups and the *H. pluvialis*-fed group had a higher CAT, SOD and peroxidase levels and anti-lipid peroxidation activity (TBARS) in the plasma and liver when compared to *S. platensis*- and *B. braunii*-fed groups. These features point to *H. pluvialis* as a feasible source of antioxidant carotenoids due their bioavailability, organ accumulation and antioxidant properties [253].

Ravi and Baskaran [254] hypothesized that the bioavailability of fucoxanthin absorbed from nanogels (NGs) was increased. Chitosan (CS) NGs were prepared using fucoxanthin (FUCO) solubilized in glycolipid (GL) or not. Fucoxanthin bioavailability was assessed after a single dose (48 h), after repeated doses for 14 days, and as dietary feeding (1 month) of CS-NG with or without GL. Regarding single (pmol/mL/48 h) and repeated dose (nmol/mL) studies, plasma FUCO was higher by 227.5% and 292.4% in CS-NGs + GL compared to CS-NGs (−GL) and control groups. Concerning the dietary study, plasma FUCO (nmol/mL) was higher by 57.5%, 400% and 287% in CS-NGs + GL compared to CS-NGs (−GL), in seaweed and control groups. Fucoxanthin was determined in plasma and tissues by HPLC. Lipid peroxidation was assessed using the TBARS technique, and was higher in the control, sequenced by FUCO + CS-NG, and then FUCO + CS-NG + GL, a result which authors attributed to the antioxidant activity protection from carotenoids. Likewise, CAT, GST and SOD activities were higher in the group FUCO + CS-NG + GL, followed by FUCO + CS-NG and with a minor concentration in the control group, corroborating that fucoxanthin could act in vivo as an antioxidant and the GL inclusion enhanced fucoxanthin bioavailability. Furthermore, the researchers also found that, beyond the well-known passive diffusion of carotenoids as the means of absorption, GL and FUCO had an agonist action on the transcriptional factor reported to regulate carotenoid absorption (PPARγ), which could also activate the membrane-bound lipid transporter SRB1 (enterocyte apical membrane transporter), positively regulating the active transport of carotenoids [254].

Campos et al. [255] evaluated the effects of lycopene on reducing the redox imbalance and inflammation induced by cigarette smoke (CS) in a murine emphysema model. During the in vivo study, 40 mice were divided into five groups: a control exposed to ambient air (CG), a vehicle-control group that received 200 μL of sunflower oil by gavage (OG), a group exposed to CS and two groups with lycopene administered (diluted in sunflower oil) at doses of either 25 or 50 mg/kg/day prior to exposure to CS (LY25 + CS and LY50 + CS). The animals were exposed three times a day to an amount of smoke equivalent to that of 12 cigarettes in a smoke chamber, and the total treatment time lasted 60 days. Animals were euthanized 24 h after the 60th day of smoke exposure. Lipid peroxidation was performed using TBARS and SOD, and GSH/GSSG ratios were used to assess antioxidant potential. SOD activity showed no difference between CS and groups with lycopene and was higher in CG and OG groups. CAT activity was higher in CS in comparison to CG and OG groups, while in both groups with lycopene, CAT was lower than in the CS group. GPx activity increased in the CS group compared to the CG and OG control groups. An increase in GPx activity in the LY25 + CS group compared to the CG, OG and CS groups was demonstrated. GPx activity was lower in the LY50 + CS group than in the LY25 + CS group. Regarding GSH/GSSG ratios, CS presented a lower ratio than all groups and LY50 + CS showed a higher ratio. There was an increase in TBARS levels in the CS group compared to the CG group. Conversely, administration of lycopene reduced TBARS levels in the LY50 + CS group compared to the CS group alone. In addition to the lycopene proven antioxidant activity, the authors also performed the analysis of histopathological features of the pulmonary parenchyma, cell influx by bronchoalveolar lavage and inflammatory cytokines from the bronchoalveolar fluid. Both lycopene-treated groups presented a histological pattern similar to the CG instead of CS group, which presented fragmented collagen fibers, alveolar septa rupture, increased airspace and other emphysema-like features. Furthermore, lycopene-treated groups presented less leukocyte influx in bronchoalveolar lavage when compared to the CS group and both lycopene-treated groups presented a reduction in inflammatory mediators (IFN-γ, TNF-α and IL-10) which were overexpressed in the CS group. Thus, besides antioxidant properties, lycopene also presented anti-inflammatory action [255].

## 6. Data Interpretation

The results using different techniques of antioxidant assays might differ among each other, both in vitro and in vivo, and can be influenced by the proper choice of the method (such as misuse of a method, designed for an in vitro experiment but extrapolated to an in vivo procedure) or by the nature of the assay, where the antioxidant in question could trigger a reductive or oxidative response.

Since it was proposed for solid food evaluation by the QUENCHER approach (substances capable of absorbing energy from a fluorophore), an antioxidant capacity assay should be able to be performed quickly, easily, cheaply and be reproducible [256]; therefore, many described methods were adapted to this approach (ABTS, DPPH, ORAC, FRAP among others) [257,258,259,260]. Despite the advantages and previous applications of the QUENCHER methods, these assays are not yet extensively used, probably due to a lack of validation studies of these methodologies [257]. Another feature of an in vitro assay is that it should be fairly correlated with in vivo assays. In vivo methods are usually used in combination with other methods to corroborate the findings in studies, since some aspects might be misinterpreted or lead to false results by the reactions in biological systems.

Other studies demonstrate the total concentration of an endogenous in vivo antioxidant as a single marker of antioxidant activity. Nevertheless, some in vivo antioxidants might be enhanced in stress situations and/or act as pro-oxidants instead. The targeting of the oxidation is discussed, with some considering it as an aggravating factor, while other situations present these species as targeting specific deleterious molecules/cells.

The protective effects of SOD have been demonstrated in animal models, where SOD significantly correlates with the protection of the heart and brain from ischemic injury [261,262]. The down regulation of SOD in diabetic mice correlated to escalation of diabetic nephropathy, while the treatment with mimetic SOD ameliorated these changes [263]. The effectiveness of SOD3 application has been proven in the treatment of skin infections in in vivo and in vitro studies, acting as a regulator of signaling cells and inflammatory cytokines [264,265]. However, SOD overexpression is associated with oxidative stress, verified by increased lipid peroxidation in cell models presenting high levels of CuZnSOD [266]; along with that, another model in cell culture also demonstrated that CuZnSOD overexpression results in hypersensitivity to oxidative stress, which could be compensated by increased catalase [267] or GPx levels [268]. In addition, in mouse tumor cells, SOD3 overexpression increased vessel length and diameter, attenuating hypoxia, resulting in better chemotherapy prognosis [201]. High levels of lipid peroxidation are associated with Down syndrome, where the oxidative stress in this population might be due to elevated CuZnSOD activity [269,270]. Similarly, higher concentrations of SOD and GPx were found in people with coronary artery disease (CAD), which had a higher pro-oxidant-antioxidant balance [271]. SOD1, GSH and GPx were elevated in patients with multiple myeloma (MM) and increased resistance to bortezomib, the reference drug. The patients that presented this condition showed poor prognosis for MM [272]. Correspondingly, CAT expression can be enhanced in response to acute stress, as in the presence of the oxidation intermediate H_2_O_2_ [273,274,275,276].

Despite the controversial applications and methods of the GSH/GSSG ratio being applied, the importance of this ratio in identifying stressful situations has proven it to be valid [172,277]. Thus, the ratio GSH/GSSG could be used to evaluate oxidative stress.

Bioavailability of bioactive compounds is defined as the proportion of an antioxidant that is digested, absorbed, and utilized in normal metabolism, which can be an issue, since the absorption and transport processes of many of these compounds are complex and not fully understood [231]. Several issues must be considered in the evaluation of the bioavailability of antioxidant compounds. One of the most important issues to address is the food matrix, since bioaccessibility is directly linked to it, thus affecting the bioavailability [278]. Aside from that, chemical interactions with other phytochemicals and biomolecules present in food interfere in the bioavailability of antioxidants [225,231].

While vitamins and carotenoids are usually assessed in isolated forms, several polyphenols are assessed in combination with other compounds, or within the food matrix (as described above in guarana, tea and coffee studies). Therefore, the studies should be clear to state whether the food matrix improves or reduces the bioavailability of the compound in question. The use of a lipid medium to evaluate carotenoids strongly augments the bioavailability of this compound [249], while the aqueous medium enhances the bioavailability of ascorbic acid, as it is a water-soluble vitamin [279]. Furthermore, the assessment of more than one antioxidant at a time could influence the results of bioavailability. Ascorbic acid is known to reduce vitamin E phenoxyl radicals, recycling vitamin E [280]; while this feature enhances vitamin E levels, it lowers ascorbate levels [281]. To correctly assess the bioavailability of bioactive compounds, a few points should be taken into consideration. Overall, the bioavailability of many bioactive compounds has been underestimated; since some metabolites might have been ignored, some methods need to be optimized and the conservation of samples that could be degraded under frozen storage should be performed. Moreover, other aspects might modify bioavailability, such as the interaction and modification of gut microbiota. The sample must be assessed in the proper site; for example, most studies collect urine to determine bioavailability and some compounds are excreted in bile preferentially. Additionally, personal differences in individuals, such as the background diet of the studied population, can interfere in the results, which makes it difficult to compare the studies [224].

It is important to realize that, despite natural antioxidants positively affecting health status, they should not be compared or equated to pharmaceutical drug action, and, therefore, no clear pharmacological responses should be expected from them. Pharmaceutical drugs act on a specific site, such as an enzyme, a receptor or a transporter. Preferably, the action of a drug should be specific—that is, act on a unique target and induce a strong effect allowing its clinical effects to be relatively easy to measure [282]. Food and food-derived compounds have a multitude of actions that are not specific and their effects on health are not easy to determine [283], and therefore, this extrapolation is erroneous.

## 7. Conclusions

Antioxidant assessment in vitro and in vivo are largely used to determine the antioxidant capacity in diverse foods and biological samples. However, due to the lack of method and interpretation standardization, there are non-comparable results and untrustworthy information. The careful choice of method, and its proper application, are fundamental to achieving reliable results.

Regarding antioxidant activity methodologies that are widely used, the ORAC method has the most applicable correlation to food intake in the database; however, this assay is an expensive method, while other methods, such as ABTS, have related results with minor costs. When antioxidant activity is performed in in vivo assays, the isolated use of a method can be controversial. Diverse techniques have results that correlate with both the antioxidant protective status and the stress markers. The bioavailability of secondary metabolites or bioactive compounds also needs to be analyzed properly. It is important to evaluate the kinetics of the substance to successfully determine the site of sample collection and analysis, as well the use of adequate methods for these determinations.

## Figures and Tables

**Table 1:** Advantages and disadvantages of DPPH, ABTS, ORAC and FRAP, TRAP methods.

Method	Advantages	Disadvantages
DPPH	Easy procedure Low cost Fast reaction	EC50 concept could be hard to interpret No standardization among time to assess or endpoint reaction
ABTS	Low cost Fast reaction Can be used to assess pH effect on activity, since is stable pH	Now standardized, which difficult comparison among results It is necessary an extra step to generate free radical from ABTS salt The generated free radical is not stable for long periods of time
ORAC	Once the method is standardized allows comparison Takes in account both time and antioxidant reaction Use biologically relevant antioxidants	Expensive pH-sensitive Quantification of results takes long time Results can vary across different equipment
FRAP	Used in samples of plant origin.	Uses high volume of reagent.
TRAP	It is sensitive to all antioxidants present in the sample.	It is a complex and time-consuming technique that requires a high degree of knowledge and experience.

Adapted from Zulueta et al. [138].

**Table 2:** Summary of studies on bioavailability and action of polyphenols, vitamins and carotenoids using animal models or clinical trials.

Compound	Study Design	Bioavailability	Assays	Results	Related Antioxidant Activity	Reference
Guaraná powdered seed (*Paullinia cupana*)	Humans (healthy overweight adults) *n* = 12	Detected 1 h after intake and remained after plasma clearance (plasma HPLC)	ORAC	↑ORAC	Guaraná is a rich source of bioavailable catechins and contributes to reducing the oxidative stress parameters of clinically health overweight individuals by direct antioxidant action and up-regulation of antioxidant enzymes.	Yonekura et al. (2016) [178]
	Single dose (3 g/90 mg catechins and 60 mg epicatechins equivalent) daily for 15 days		SOD/CAT/GPx activities	↑CAT/GPx ↔SOD		
	Blood samples: overnight-fasting and 1 h after intake		Ex vivo LDL oxidation/H_2_O_2_ induced DNA damage in lymphocytes (Comet Assay)	↓LDL oxidation (only in the first day of study) ↓DNA damage (only after 1 h intake)		
Green (GT) and Black Tea (BT) (commercially acquired)	Male Wistar rats (n = 18)	Not evaluated	Drug metabolizing enzymes activity (hepatic and pulmonary)	↑P450 (CYP) 1A1 (hep) ↑UDP-glucoronosyltransferase (hep/pulm) ↑CYP 1A2 (hep) ↓CYP 2C (hep) ↓CYP 2E1 (hep) ↓CYP 3A (hep)	Feeding both tea drinks to rats modulated drug metabolizing enzymes at a transcriptional level and reduced oxidative stress in the liver and lungs, but green tea was more effective in reducing oxidative stress. Their possible interactions with drugs or toxic compounds should be taken into account.	Yao et al. (2014) [179]
	Ad libitum with food and as water replacement for 5 weeks		GSH, GSSG and GSH/GSSG ratio	↓[GSH] (hep/pulm) ↓Lipid peroxide (pulm/GT)		
	Blood and tissue (liver and lungs) samples after being euthanized		GPx and GSR TBARS and ROS			
			DNA-binding activity of nuclear factors			
Coffee (caffeic and ferulic acids)	Humans (n = 20)	Detected 1 h after intake (plasma HPLC)	ORAC	↑ORAC		Lara-Guzmán et al. (2016) [180]
			FRAP/TRAP	↑FRAP/↔TRAP	The experiments on plasma with caffeic and ferulic acids showed a significant increase in the antioxidant activity as well as delay of LDL oxidation.	
	After acute consumption (1 h/400 mL)		Ex vivo LDL oxidation	↓LDL oxidation		
Vitamin C supplementation	Male Sprague Dawley rats (*n* = 6–7 per group)	Detected in plasma and cerebral tissue (spectrophotometry)	Histopathology	Diabetic state: ↑infarct volume and edema Vit. C: ↓damage	Daily intake of ascorbic acid attenuates the exacerbation of cerebral ischemic injury in a diabetic state, which may be attributed to anti-apoptotic and anti-inflammatory effects via the improvement of augmented oxidative stress in the brain. Ascorbic acid supplementation may protect endothelial function against the exacerbated ischemic oxidative injury and improve its transport through SVCT2 in the cortex.	Iwata et al. (2014) [184]
	Streptozotocin-induced type 1 diabetes		IHC (SVCT2, GLUT-1, cleaved caspase-3, TNF-α, IL-1β	↓cleaved caspase-3 ↓inflammatory cytokines (TNF-α and IL-1β)		
	100 mg/kg of ascorbic acid (gavage) for 2 weeks before cerebral ischemia-reperfusion protocol		PCR (SVCT2 and GLUT1)	↑SVCT2 (neurons and endothelial cells) ↑GLUT1 (endothelial cells)		
	Cerebral ischemia-reperfusion (infarct induction)		Superoxide production	↓superoxide radical		
Multivitamin supplementation (vit. C + vit. E + sodium selenite + β-carotene)	Female Sprague Dawley rats (*n* = 40)	Not evaluated	Histopathology	↓honeycomb-like injury ↓edema ↔inflammatory infiltrate	The antioxidant combination protected lung tissue against damage by enhancing biochemical parameters and pulmonary edema, while no significant effect on protection of pulmonary inflammation was observed. The antioxidant vitamin supplementation with selenium can be used in the prevention of acute lung injury.	Bayrak et al. (2016) [185]
	Vitamin C (100 mg/kg/day), vitamin E (100 mg/kg/day), sodium selenite (0.2 mg/kg/day and β-carotene (15 mg/kg/day) via gavage for 3 days before the injury protocol					
	D-galactosamine-induced (DGaIN) acute lung injury		GSH/GPx/PON TF/LDH/CAT/SOD/MPO/XO /	↑GSH/GPx/PON ↓TF/LDH/CAT/SOD/MPO/XO/Na^+^/K^+^ ATPase		
			LPO (MDA)	↓LPO		
Multivitamin supplementation (vit. C + vit. E + sodium selenite + β-carotene)	Female Sprague Dawley rats (*n* = 40)	Not evaluated	Histopathology	↓edema ↓necrosis ↓inflammatory infiltrate	The combination of antioxidants suppressed histopathological changes in the liver and biochemical parameters in D-GaIN-induced hepatotoxicity rats. The antioxidant vitamin supplementation with selenium can be used in the prevention of acute hepatotoxicity.	Catal et al. (2017) [186]

	Vitamin C (100 mg/kg/day), vitamin E (100 mg/kg/day), sodium selenite (0.2 mg/kg/day and β-carotene (15 mg/kg/day) via gavage for 3 days before the injury protocol		Blood GSH/CAT Blood AST/ALT/ALP/GGT/LDH Blood sialic and uric acid Hepatic CAT/SOD/GSH/GPx Hepatic GST	↑blood GSH and CAT ↓blood AST/ALT/ALP/GGT/LDH ↓blood sialic and uric acid ↑hepatic CAT/SOD/GSH/GPx ↔hepatic GST		
	D-galactosamine-induced (DGaIN) acute liver injury		LPO (MDA)	↓hepatic LPO		
			Histopathology			
Carotenoids derived from microalgal biomass (*Spirulina platensis*, *Haematococcus pluvialis* and *Botryococcus braunii*)	Male Wistar rats (*n* = 25)	Detected in plasma after 2 h, in the liver after 4 h and in the eyes after 6 h (HPLC/LC-MS) Carotenoid’s accumulation: liver > eyes > plasmaAstaxanthin from *H. pluvialis* had better bioavailability and accumulation than other groups.	Plasma and hepatic SOD, CAT, peroxidase and lipid peroxidation (TBARS)	↑plasma and hepatic SOD, CAT and peroxidase ↓Lipid peroxidation		Rao et al. (2013) [194]
	Sigle dose of microalgal biomass (200 µM equivalent of β-carotene, astaxanthin and lutein) via gavage for 15 days				These results indicate that the astaxanthin from *H. pluvialis* has better bioavailability and better antioxidant properties compared to other carotenoids. Microalgae biomass is capable of preventing lipid peroxidation through scavenging free radicals and hydroxyl radicals in living cells and also restoring antioxidant enzyme activity.	
Fucoxanthin (FUCO) solubilized in glycolipid (GL) and absorbed from chitosan nanogels (NG)	Rat model Groups: control, FUCO, FUCO+NG-GL, FUCO+NG+GL	Plasma FUCO (HPLC). After single dose: ↑ 227.5% After repeated doses: ↑ 292.4% (compared to control and -GL groups).	CAT/GST/SODLipid peroxidation (TBARS)	↑CAT/GST/SOD (+GL > ☐GL > controls) ↓Lipid peroxidation (controls > ☐GL > +GL)	The advantage of CS-NGs + GL for improved FUCO bioavailability via passive and active transport through PPARc mediated SRB1 activation was demonstrated. Elevated plasma and tissue levels of FUCO in these groups could be the reason for a higher activity of antioxidant enzymes and lower lipid peroxides.	Ravi and Baskaram (2017) [195]
	Single dose study: 48 h Repeated dose study: 14 days Dietary feeding study: 1 month	Plasma FUCO (HPLC) after dietary feeding: ↑ 57.5% (compared to ☐GL) ↑ 400% (compared to seaweed) ↑ 287% (compared to control)	PPARγ/SRB1	↑PPARγ/SRB1(FUCO and GL had an agonist action)		
Lycopene	Murine emphysema model (*n* = 40)	Not evaluated	Histopathology	Inhibited emphysema-like features when treated with LY	Lycopene acts as an antioxidant and anti-inflammatory through the neutralization of reactive species production in vitro and in vivo, the restoration of the GSH/GSSG ratio, decreasing oxidative damage, decreasing pro-inflammatory cytokines through decreased cell influx and the direct suppression of cytokine production.	Campos et al. (2017) [196]
	Cigarette smoke exposure 3 times a day for 60 days		SOD/CAT/GPxGSH/GSSG ratio	↔SOD/↓CAT/↑GPx ↑GSH/GSSG ratio		
	Treatment with lycopene (LY) diluted in sunflower oil (25 and 50 mg/kg/day)		TBARS	↓TBARS		
			IFN-γ/TNF-α/IL-10 (bronchoalveolar fluid)	↓ IFN-γ/TNF-α/IL-10		
			Leukocyte influx (bronchoalveolar fluid)	↓ Leukocyte influx		

## Data Availability

Data sharing not applicable. No new data were created or analyzed in this study. Data sharing is not applicable to this article.

## References

[1] Amorati R., Valgimigli L. (2018). Methods to Measure the Antioxidant Activity of Phytochemicals and Plant Extracts. J. Agric. Food Chem..

[2] Xu D.P., Li Y., Meng X., Zhou T., Zhou Y., Zheng J., Zhang J.J., Li H.B. (2017). Natural Antioxidants in Foods and Medicinal Plants: Extraction, Assessment and Resources. Int. J. Mol. Sci..

[3] Liaudanskas M., Žvikas V., Petrikaitė V. (2021). The Potential of Dietary Antioxidants from a Series of Plant Extracts as Anticancer Agents against Melanoma, Glioblastoma, and Breast Cancer. Antioxidants.

[4] Lu Q.Y., Summanen P.H., Lee R.P., Huang J., Henning S.M., Heber D., Finegold S.M., Li Z. (2017). Prebiotic Potential and Chemical Composition of Seven Culinary Spice Extracts. J. Food Sci..

[5] Fu L., Xu B.T., Gan R.Y., Zhang Y., Xu X.R., Xia E.Q., Li H.B. (2011). Total phenolic contents and antioxidant capacities of herbal and tea infusions. Int. J. Mol. Sci..

[6] Deng G.F., Xu X.R., Guo Y.J., Xia E.Q., Li S., Wu S., Chen F., Ling W.H., Li H.B. (2012). Determination of antioxidant property and their lipophilic and hydrophilic phenolic contents in cereal grains. J. Funct. Foods.

[7] Cömert E.D., Gökmen V. (2018). Evolution of food antioxidants as a core topic of food science for a century. Food Res. Int..

[8] Thaipong K., Boonprakob U., Crosby K., Cisneros-Zevallos L., Hawkins Byrne D. (2006). Comparison of ABTS, DPPH, FRAP, and ORAC assays for estimating antioxidant activity from guava fruit extracts. J. Food Compos. Anal..

[9] Harasym J., Oledzki R. (2014). Effect of fruit and vegetable antioxidants on total antioxidant capacity of blood plasma. Nutrition.

[10] Gulcin İ. (2020). Antioxidants and antioxidant methods: An updated overview. Arch. Toxicol..

[11] Sunkara A., Raizner A. (2019). Supplemental Vitamins and Minerals for Cardiovascular Disease Prevention and Treatment. Methodist Debakey Cardiovasc. J..

[12] López-Alarcón C., Denicola A. (2013). Evaluating the antioxidant capacity of natural products: A review on chemical and cellular-based assays. Anal. Chim. Acta.

[13] Pisoschi A.M., Pop A. (2015). The role of antioxidants in the chemistry of oxidative stress: A review. Eur. J. Med. Chem..

[14] Benzie I.F.F., Wachtel-Galor S. (2012). Increasing the antioxidant content of food: A personal view on whether this is possible or desirable. Int. J. Food Sci. Nutr..

[15] Guo Q., Li F., Duan Y., Wen C., Wang W., Zhang L., Huang R., Yin Y. (2020). Oxidative stress, nutritional antioxidants and beyond. Sci. China Life Sci..

[16] Abramovič H., Grobin B., Ulrih N.P., Cigić B. (2018). Relevance and Standardization of In vitro Antioxidant Assays: ABTS, DPPH, and Folin–Ciocalteu. J. Chem..

[17] Liu S., Wang Y. (2015). Mass spectrometry for the assessment of the occurrence and biological consequences of DNA adducts. Chem. Soc. Rev..

[18] Apak R. (2019). Current Issues in Antioxidant Measurement. J. Agric. Food Chem..

[19] Apak R., Özyürek M., Güçlü K., Çapanoğlu E. (2016). Antioxidant Activity/Capacity Measurement. 2. Hydrogen Atom Transfer (HAT)-Based, Mixed-Mode (Electron Transfer (ET)/HAT), and Lipid Peroxidation Assays. J. Agric. Food Chem..

[20] Fraga C.G., Oteiza P.I., Galleano M. (2014). In vitro measurements and interpretation of total antioxidant capacity. Biochim Biophys Acta..

[21] Istas G., Wood E., Le Sayec M., Rawlings C., Yoon J., Dandavate V., Cera D., Rampelli S., Costabile A., Fromentin E. (2019). Effects of aronia berry (poly)phenols on vascular function and gut microbiota: A double-blind randomized controlled trial in adult men. Am. J. Clin. Nutr..

[22] Lavefve L., Howard L.R., Carbonero F. (2020). Berry polyphenols metabolism and impact on human gut microbiota and health. Food Funct..

[23] Yan Z., Zhong Y., Duan Y., Chen Q., Li F. (2020). Antioxidant mechanism of tea polyphenols and its impact on health benefits. Anim. Nutr..

[24] Amengual J. (2019). Bioactive Properties of Carotenoids in Human Health. Nutrients.

[25] Pérez-Gálvez A., Viera I., Roca M. (2020). Carotenoids and Chlorophylls as Antioxidants. Antioxidants.

[26] Fiedor J., Burda K. (2014). Potential Role of Carotenoids as Antioxidants in Human Health and Disease. Nutrients.

[27] Sharkey I. (2012). Advances in photosynthesis and respiration. Photosynth. Res..

[28] Nisar N., Li L., Lu S., Khin N.C., Pogson B.J. (2015). Carotenoid Metabolism in Plants. Plant Metab. Synth. Biol..

[29] Widomska J., Gruszecki W.I., Subczynski W.K. (2021). Factors Differentiating the Antioxidant Activity of Macular Xanthophylls in the Human Eye Retina. Antioxidants.

[30] Duda M., Cygan K., Wisniewska-Becker A. (2020). Effects of Curcumin on Lipid Membranes: An EPR Spin-label Study. Cell Biochem. Biophys..

[31] Vona R., Gambardella L., Cittadini C., Straface E., Pietraforte D. (2019). Biomarkers of Oxidative Stress in Metabolic Syndrome and Associated Diseases. Oxid. Med. Cell Longev..

[32] Förstermann U., Xia N., Li H. (2017). Roles of Vascular Oxidative Stress and Nitric Oxide in the Pathogenesis of Atherosclerosis. Circ. Res..

[33] Blesa J., Trigo-Damas I., Quiroga-Varela A., Jackson-Lewis V.R. (2015). Oxidative stress and Parkinson’s disease. Front. Neuroanat..

[34] Wang X., Wang W., Li L., Perry G., Lee H., Zhu X. (2014). Oxidative stress and mitochondrial dysfunction in Alzheimer’s disease. Biochim. Biophys. Acta.

[35] Rodriguez-Amaya D.B. (2015). Status of carotenoid analytical methods and in vitro assays for the assessment of food quality and health effects. Curr. Opin. Food Sci..

[36] Arunkumar R., Gorusupudi A., Bernstein P.S. (2020). The macular carotenoids: A biochemical overview. Biochim. Biophys. Acta Mol. Cell Biol. Lipids.

[37] Nimse S.B., Pal D. (2015). Free radicals, natural antioxidants, and their reaction mechanisms. RSC Adv..

[38] Chantrell S.J., McAuliffe C.A., Munn R.W., Pratt A.C., Land E.J. (1977). Excited states of protoporphyrin IX dimethyl ester: Reaction on the triplet with carotenoids. J. Chem. Soc. Faraday Trans. Phys. Chem. Condens. Phases.

[39] Jeong Y., Lim J.W., Kim H. (2019). Lycopene Inhibits Reactive Oxygen Species-Mediated NF-κB Signaling and Induces Apoptosis in Pancreatic Cancer Cells. Nutrients.

[40] Heymann T., Heinz P., Glomb M.A. (2015). Lycopene inhibits the isomerization of β-carotene during quenching of singlet oxygen and free radicals. J. Agric. Food Chem..

[41] Saini R.K., Keum Y.S. (2018). Carotenoid extraction methods: A review of recent developments. Food Chem..

[42] Moran N.A., Jarvik T. (2010). Lateral transfer of genes from fungi underlies carotenoid production in aphids. Science.

[43] Saini R.K., Nile S.H., Park S.W. (2015). Carotenoids from fruits and vegetables: Chemistry, analysis, occurrence, bioavailability and biological activities (Part 3). Food Res. Int..

[44] Shah M.M.R., Liang Y., Cheng J.J., Daroch M. (2016). Astaxanthin-producing green microalga Haematococcus pluvialis: From single cell to high value commercial products. Front. Plant Sci..

[45] Grosso C., Valentão P., Ferreres F., Andrade P.B. (2015). Alternative and efficient extraction methods for marine-derived compounds. Mar. Drugs.

[46] Singh A., Ahmad S., Ahmad A. (2015). Green extraction methods and environmental applications of carotenoids-a review. RSC Adv..

[47] Singh D., Barrow C.J., Mathur A.S., Tuli D.K., Puri M. (2015). Optimization of zeaxanthin and β-carotene extraction from Chlorella saccharophila isolated from New Zealand marine waters. Biocatal. Agric. Biotechnol..

[48] Saini R.K., Keum Y.-S. (2017). Progress in microbial carotenoids production. Indian J. Microbiol..

[49] Alfonsi K., Colberg J., Dunn P.J., Fevig T., Jennings S., Johnson T.A., Stefaniak M. (2008). Green chemistry tools to influence a medicinal chemistry and research chemistry based organisation. Green Chem..

[50] Capello C., Fischer U., Hungerbühler K. (2007). What is a green solvent? A comprehensive framework for the environmental assessment of solvents. Green Chem..

[51] Jiang Q. (2014). Natural forms of vitamin E: Metabolism, antioxidant, and anti-inflammatory activities and their role in disease prevention and therapy. Free Radic. Biol. Med..

[52] Casadesús A., Arabia A., Pujolriu R., Munné-Bosch S. (2020). Differential accumulation of tocochromanols in photosynthetic and non-photosynthetic tissues of strawberry plants subjected to reiterated water deficit. Plant Physiol. Biochem..

[53] Drotleff A.M., Bohnsack C., Schneider I., Hahn A., Ternes W. (2014). Human oral bioavailability and pharmacokinetics of tocotrienols from tocotrienol-rich (tocopherol-low) barley oil and palm oil formulations. J. Funct. Foods.

[54] Azzi A. (2019). Tocopherols, tocotrienols and tocomonoenols: Many similar molecules but only one vitamin E. Redox Biol..

[55] McLaughlin P.J., Weihrauch J.L. (1979). Vitamin E content of foods. J. Am. Diet. Assoc..

[56] Birringer M., Lorkowski S. (2019). Vitamin E: Regulatory role of metabolites. IUBMB Life.

[57] Aune D., Keum N., Giovannucci E., Fadnes L.T., Boffetta P., Greenwood D.C., Tonstad S., Vatten L.J., Riboli E., Norat T. (2018). Dietary intake and blood concentrations of antioxidants and the risk of cardiovascular disease, total cancer, and all-cause mortality: A systematic review and dose-response meta-analysis of prospective studies. Am. J. Clin. Nutr..

[58] Mangge H., Becker K., Fuchs D., Gostner J.M. (2014). Antioxidants, inflammation and cardiovascular disease. World J. Cardiol..

[59] Liakopoulos V., Roumeliotis S., Zarogiannis S., Eleftheriadis T., Mertens P.R. (2019). Oxidative stress in hemodialysis: Causative mechanisms, clinical implications, and possible therapeutic interventions. Semin. Dial..

[60] Nicod N., Parker R.S. (2013). Vitamin E Secretion by Caco-2 Monolayers to APOA1, but Not to HDL, Is Vitamer Selective. J. Nutr..

[61] Szewczyk K., Chojnacka A., Górnicka M. (2021). Tocopherols and Tocotrienols-Bioactive Dietary Compounds; What Is Certain, What Is Doubt?. Int. J. Mol. Sci..

[62] Wong W.Y., Ward L.C., Fong C.W., Yap W.N., Brown L. (2017). Anti-inflammatory γ- and δ-tocotrienols improve cardiovascular, liver and metabolic function in diet-induced obese rats. Eur. J. Nutr..

[63] Wong S.Y., Teo J.S.M., Chai S.F., Yeap S.L., Lau A.J. (2019). Vitamin E analogues differentially inhibit human cytochrome P450 3A (CYP3A)-mediated oxidative metabolism of lithocholic acid: Impact of δ-tocotrienol on lithocholic acid cytotoxicity. Toxicology.

[64] Arrozi A.P., Shukri S.N.S., Ngah W.Z.W., Yusof Y.A.M., Damanhuri M.H.A., Jaafar F., Makpol S. (2020). Comparative effects of alpha-and gamma-tocopherol on mitochondrial functions in Alzheimer’s Disease in vitro model. Sci. Rep..

[65] Pingret D., Fabiano-Tixier A.-S., Chemat F. (2013). Degradation during application of ultrasound in food processing: A review. Food Control.

[66] Han R.M., Zhang J.P., Skibsted L.H. (2012). Reaction Dynamics of Flavonoids and Carotenoids as Antioxidants. Molecules.

[67] Zerbinati C., Caponecchia L., Fiori C., Sebastianelli A., Salacone P., Ciacciarelli M., Iuliano L. (2020). Alpha- and gamma-tocopherol levels in human semen and their potential functional implications. Andrologia.

[68] De Andrade Lima M., Kestekoglou I., Charalampopoulos D., Chatzifragkou A. (2019). Supercritical Fluid Extraction of Carotenoids from Vegetable Waste Matrices. Molecules.

[69] Oliver J., Palou A. (2000). Chromatographic determination of carotenoids in foods. J. Chromatogr. A.

[70] Xu Z. (2008). Comparison of extraction methods for quantifying vitamin E from animal tissues. Bioresour. Technol..

[71] Pawlowska E., Szczepanska J., Blasiak J. (2019). Pro- and Antioxidant Effects of Vitamin C in Cancer in correspondence to Its Dietary and Pharmacological Concentrations. Oxid. Med. Cell Longev..

[72] Wang L., Gao Y., Li J., Subirade M., Song Y., Liang L. (2016). Effect of resveratrol or ascorbic acid on the stability of α-tocopherol in O/W emulsions stabilized by whey protein isolate: Simultaneous encapsulation of the vitamin and the protective antioxidant. Food Chem..

[73] May J.M. (2012). Vitamin C transport and its role in the central nervous system. Subcell Biochem..

[74] Spector R., Johanson C.E. (2014). The nexus of vitamin homeostasis and DNA synthesis and modification in mammalian brain. Mol. Brain.

[75] Kaufman S. (1966). Coenzymes and hydroxylases: Ascorbate and dopamine-beta-hydroxylase; tetrahydropteridines and phenylalanine and tyrosine hydroxylases. Pharmacol. Rev..

[76] Kaźmierczak-Barańska J., Boguszewska K., Adamus-Grabicka A., Karwowski B.T. (2020). Two Faces of Vitamin C-Antioxidative and Pro-Oxidative Agent. Nutrients.

[77] Du J., Cullen J.J., Buettner G.R. (2012). Ascorbic acid: Chemistry, biology and the treatment of cancer. Biochim. Biophys. Acta.

[78] Carr A.C., Maggini S. (2017). Vitamin C and Immune Function. Nutrients.

[79] Nowak D. (2021). Vitamin C in Human Health and Disease. Nutrients.

[80] Salaj N., Kladar N., Čonić B.S., Jeremić K., Hitl M., Gavarić N., Božin B. (2021). Traditional multi-herbal formula in diabetes therapy—Antihyperglycemic and antioxidant potential. Arab. J. Chem..

[81] Farag R.S., Abdel-Latif M.S., Abd El Baky H.H., Tawfeek L.S. (2020). Phytochemical screening and antioxidant activity of some medicinal plants’ crude juices. Biotechnol. Rep..

[82] Sarmiento-Salinas F.L., Perez-Gonzalez A., Acosta-Casique A., Ix-Ballote A., Diaz A., Treviño S., Rosas-Murrieta N.H., Millán-Perez-Peña L., Maycotte P. (2021). Reactive oxygen species: Role in carcinogenesis, cancer cell signaling and tumor progression. Life Sci..

[83] Ghafoor K., Juhaimi F.A.M., Özcan M., Uslu N., Babiker E.E., Ahmed I.A.M. (2020). Total phenolics, total carotenoids, individual phenolics and antioxidant activity of ginger (*Zingiber officinale*) rhizome as affected by drying methods. LWT.

[84] Mwamatope B., Tembo D., Chikowe I., Kampira E., Nyirenda C. (2020). Total phenolic contents and antioxidant activity of Senna singueana, Melia azedarach, Moringa oleifera and Lannea discolor herbal plants. Sci. Afr..

[85] Yu M., Gouvinhas I., Rocha J., Barros A.I.R.N.A. (2021). Phytochemical and antioxidant analysis of medicinal and food plants towards bioactive food and pharmaceutical resources. Sci. Rep..

[86] Yousfi F., Abrigach F., Petrovic J.D., Sokovic M., Ramdani M. (2021). Phytochemical screening and evaluation of the antioxidant and antibacterial potential of Zingiber officinale extracts. S. Afr. J. Bot..

[87] Zhang J., Chen J., Liang Z., Zhao C. (2014). New lignans and their biological activities. Chem. Biodivers..

[88] McDougall G.J. (2017). Phenolic-enriched foods: Sources and processing for enhanced health benefits. Proc. Nutr. Soc..

[89] Santhakumar A.B., Battino M., Alvarez-Suarez J.M. (2018). Dietary polyphenols: Structures, bioavailability and protective effects against atherosclerosis. Food Chem. Toxicol..

[90] Dei Cas M., Ghidoni R. (2019). Dietary Curcumin: Correlation between Bioavailability and Health Potential. Nutrients.

[91] Sheng H., Sun X., Yan Y., Yuan Q., Wang J., Shen X. (2020). Metabolic Engineering of Microorganisms for the Production of Flavonoids. Front. Bioeng. Biotechnol..

[92] Zakaryan H., Arabyan E., Oo A., Zandi K. (2017). Flavonoids: Promising natural compounds against viral infections. Arch. Virol..

[93] Wang T., Li Q., Bi K. (2018). Bioactive flavonoids in medicinal plants: Structure, activity and biological fate. Asian J. Pharm. Sci..

[94] Panche N.A., Diwan A.D., Chandra S.R. (2016). Flavonoids: An overview. J. Nutr. Sci..

[95] Fujiwara Y., Kono M., Ito A., Ito M. (2018). Anthocyanins in perilla plants and dried leaves. Phytochemistry.

[96] Ozcan T., Akpinar-Bayizit A., Yilmaz-Ersan L., Delikanli B. (2014). Phenolics in Human Health. Int. J. Chem. Eng. Appl..

[97] Shahidi F., Ambigaipalan P. (2015). Phenolics and polyphenolics in foods, beverages and spices: Antioxidant activity and health effects- A review. J. Funct. Foods.

[98] Gerstenmeyer E., Reimer S., Berghofer E., Schwartz H., Sontag G. (2013). Effect of thermal heating on some lignans in flax seeds, sesame seeds and rye. Food Chem..

[99] De Freitas V., Mateus N. (2012). Protein/Polyphenol Interactions: Past and Present Contributions. Mechanisms of Astringency Perception. Cur. Org. Chem..

[100] Smeriglio A., Barreca D., Bellocco E., Trombetta D. (2017). Proanthocyanidins and hydrolysable tannins: Occurrence, dietary intake and pharmacological effects. Br. J. Pharmacol..

[101] Bianchi S., Kroslakova I., Janzon R., Mayer I., Saake B., Pichelin F. (2015). Characterization of condensed tannins and carbohydrates in hot water bark extracts of European softwood species. Phytochemistry.

[102] Kumar S., Pandey A.K. (2013). Chemistry and Biological Activities of Flavonoids: An Overview. Sci. World J..

[103] Carbonell-Capella J.M., Buniowska M., Barba F.J., Esteve M.J., Frígola A. (2014). Analytical Methods for Determining Bioavailability and Bioaccessibility of Bioactive Compounds from Fruits and Vegetables: A Review. Compr. Rev. Food Sci. Food Saf..

[104] Singh B., Singh J.P., Kaur A., Singh N. (2017). Phenolic composition and antioxidant potential of grain legume seeds: A review. Food Res. Int..

[105] Kim M.Y., Yoon N., Lee Y.J., Woo K.S., Kim H.Y., Lee J., Jeong H.S. (2020). Influence of Thermal Processing on Free and Bound Forms of Phenolics and Antioxidant Capacity of Rice Hull (*Oryza sativa* L.). Prev. Nutr. Food Sci..

[106] Kwatra B. (2020). A review on potential properties and therapeutic applications of grape seed extract. World J. Pharm. Res..

[107] Khoddami A., Wilkes A.M., Roberts H.T. (2013). Techniques for Analysis of Plant Phenolic Compounds. Molecules.

[108] Folin O., Ciocalteu V. (1927). On Tyrosine and Tryptophane Determinations in Proteins. J. Biol. Chem..

[109] Sasikumar J.M., Erba O., Egigu M.C. (2020). In vitro antioxidant activity and polyphenolic content of commonly used spices from Ethiopia. Heliyon.

[110] Bunzel M., Schendel R.R. (2017). Determination of (total) phenolics and antioxidant capacity in food and ingredients. Food Analysis.

[111] Singleton V.L., Rossi J.A. (1965). Colorimetry of Total Phenolics with Phosphomolybdic-Phosphotungstic Acid Reagents. Am. J. Enol. Vitic..

[112] Bibi Sadeer N., Montesano D., Albrizio S., Zengin G., Mahomoodally M.F. (2020). The Versatility of Antioxidant Assays in Food Science and Safety-Chemistry, Applications, Strengths, and Limitations. Antioxidants.

[113] Ionita P. (2021). The Chemistry of DPPH·Free Radical and Congeners. Int. J. Mol. Sci..

[114] Blois M.S. (1958). Antioxidant Determinations by the Use of a Stable Free Radical. Nature.

[115] Sirivibulkovit K., Nouanthavong S., Sameenoi Y. (2018). Paper-based DPPH Assay for Antioxidant Activity Analysis. Anal. Sci..

[116] Zorzi M., Gai F., Medana C., Aigotti R., Morello S., Peiretti P.G. (2020). Bioactive Compounds and Antioxidant Capacity of Small Berries. Foods.

[117] Ali Y.M., Kadir A.A., Ahmad Z., Yaakub H., Zakaria Z.A., Abdullah M.N. (2012). Free radical scavenging activity of conjugated linoleic acid as single or mixed isomers. Pharm. Biol..

[118] Triantis T.M., Yannakopoulou E., Nikokavoura A., Dimotikali D., Papadopoulos K. (2007). Chemiluminescent studies on the antioxidant activity of amino acids. Anal. Chim. Acta.

[119] Brand-Williams W., Cuvelier M.E., Berset C. (1995). Use of a free radical method to evaluate antioxidant activity. LWT Food Sci. Technol..

[120] Koch W., Kukuła-Koch W., Czop M., Helon P., Gumbarewicz E. (2020). The Role of Extracting Solvents in the Recovery of Polyphenols from Green Tea and Its Antiradical Activity Supported by Principal Component Analysis. Molecules.

[121] Ahmad N.A., Jumbri K., Ramli A., Abd Ghani N., Ahmad H., Lim J.W. (2018). A Kinetic Approach of DPPH Free Radical Assay of Ferulate-Based Protic Ionic Liquids (PILs). Molecules.

[122] Munteanu I.G., Apetrei C. (2021). Analytical Methods Used in Determining Antioxidant Activity: A Review. Int. J. Mol. Sci..

[123] Sarker U., Hossain M.M., Oba S. (2020). Nutritional and antioxidant components and antioxidant capacity in green morph Amaranthus leafy vegetable. Sci. Rep..

[124] Oldoni T.L.C., Silva R.C., Carpes S.T., Massarioli A.P., Alencar S.M. (2020). Antioxidant activity and development of one chromatographic method to determine the phenolic compounds from Agroindustrial Pomace. An. Acad. Bras. Cienc..

[125] Elgndi M.A., Filip S., Pavlíc B., Stanojkovíc T., Zizak Z., Zecovic Z. (2017). Antioxidative and cytotoxic activity of essential oils and extracts of *Satureja montana* L. Coriandrum sativum L. and Ocimum basilicum L. obtained by supercritical fluid extraction. J. Supercrit. Fluids.

[126] Mishra K., Ojha H., Chaudhury N.K. (2012). Estimation of antiradical properties of antioxidants using DPPH assay: A critical review and results. Food Chem..

[127] Lahouar L., El Arem A., Ghrairi F., Chahdoura H., Ben Salem H., El Felah M., Achour L. (2014). Phytochemical content and antioxidant properties of diverse varieties of whole barley (*Hordeum vulgare* L.) grown in Tunisia. Food Chem..

[128] Jorge N., Silva A.C., Aranha C.P. (2016). Antioxidant activity of oils extracted from orange (*Citrus sinensis*) seeds. An. Acad. Bras. Cienc..

[129] Olech M., Nowak R., Nowacka N., Pecio Ł., Oleszek W., Los R., Malm A., Rzymowska J. (2015). Evaluation of rose roots, a post-harvest plantation residue as a source of phytochemicals with radical scavenging, cytotoxic, and antimicrobial activity. Ind. Crops Prod..

[130] Nowak D., Gośliński M., Wojtowicz E., Przygoński K. (2018). Antioxidant Properties and Phenolic Compounds of Vitamin C-Rich Juices. J. Food Sci..

[131] Lee S.G., Wang T., Vance T.M., Hubert P., Kim D.O., Koo S.I., Chun O.K. (2017). Validation of Analytical Methods for Plasma Total Antioxidant Capacity by Comparing with Urinary 8-Isoprostane Level. J. Microbiol. Biotechnol..

[132] Ilyasov I.R., Beloborodov V.L., Selivanova I.A., Terekhov R.P. (2020). ABTS/PP Decolorization Assay of Antioxidant Capacity Reaction Pathways. Int. J. Mol. Sci..

[133] Miilošević M.D., Marinković A.D., Petrović P., Klaus A., Nikolić M.G., Prlainović N.Ž., Cvijetić I.N. (2020). Synthesis, characterization and SAR studies of bis(imino)pyridines as antioxidants, acetylcholinesterase inhibitors and antimicrobial agents. Bioorganic Chem..

[134] Gonçalves O.H., Moreira T.F.M., De Oliveira A., Bracht L., Ineu R.P., Leimann F.V. (2020). Antioxidant Activity of Encapsulated Extracts and Bioactives from Natural Sources. Curr. Pharm. Des..

[135] Floegel A., Kim D.O., Chung S.J., Koo S.I., Chun O.K. (2011). Comparison of ABTS/DPPH assays to measure antioxidant capacity in popular antioxidant-rich US foods. J. Food Compos. Anal..

[136] Tabart J., Kevers C., Pincemail J., Defraigne J.O., Dommes J. (2009). Comparative antioxidant capacities of phenolic compounds measured by various tests. Food Chem..

[137] Prior R. (2015). Oxygen radical absorbance capacity (ORAC): New horizons in relating dietary antioxidants/bioactives and health benefits. J. Funct. Foods.

[138] Zulueta A., Esteve M.J., Frígola A. (2009). ORAC and TEAC assays comparison to measure the antioxidant capacity of food products. Food Chem..

[139] Ou B., Hampsch-Woodill M., Prior R.L. (2001). Development and validation of an improved oxygen radical absorbance capacity assay using fluorescein as the fluorescent probe. J. Agric. Food Chem..

[140] Ortiz R., Antilén M., Speisky H., Aliaga M.E., López-Alarcón C., Baugh S. (2012). Application of a microplate-based ORAC-pyrogallol red assay for the estimation ofantioxidant capacity: First Action. J. AOAC Int..

[141] Ortiz R., Antilén M., Speisky H., Aliaga M.E., López-Alarcón C. (2011). Analytical parameters of the microplate-based ORAC-pyrogallol red assay. J. AOAC Int..

[142] Ou B., Chang T., Huang D., Prior R.L. (2013). Determination of total antioxidant capacity by oxygen radical absorbance capacity (ORAC) using fluorescein as the fluorescence probe: First Action 2012.23. J. AOAC Int..

[143] Wang H., Cao G., Prior R.L. (1996). Total Antioxidant Capacity of Fruits. J. Agric. Food Chem..

[144] Cao G., Prior R.L. (1999). Measurement of oxygen radical absorbance capacity in biological samples. Methods Enzymol..

[145] Yongsheng C., Gu C., Xiong F., Rui-Hai L. (2015). Phytochemical Profiles and Antioxidant Activity of Different Varieties of Adinandra Tea (Adinandra Jack). J. Agric. Food Chem..

[146] ORAC Database. http://oracdatabase.com/.

[147] Speisky H., López-Alarcón C., Gómez M., Fuentes J., Sandoval-Acuña C. (2012). First web-based database on total phenolics and oxygen radical absorbance capacity (ORAC) of fruits produced and consumed within the south Andes region of South America. J. Agric. Food Chem..

[148] Huang D., Ou B., Prior R.L. (2005). The chemistry behind antioxidant capacity assays. J. Agric. Food Chem..

[149] Wojtunik-Kulesza K.A. (2020). Approach to optimization of FRAP methodology for studies based on selected monoterpenes. Molecules.

[150] Berker K.I., Demirata B., Apak R. (2012). Determination of total antioxidant capacity of lipophilic and hydrophilic antioxidants in the same solution by using ferric-ferricyanide assay. Food Anal. Methods.

[151] Berker K.I., Güçlük K., Tor I., Demirata B., Apak R. (2010). Total antioxidant capacity assay using optimized ferricyanide/Prussian blue method. Food Anal. Methods.

[152] Maciejczyk M., Szulimowska J., Taranta-Janusz K., Werbel K., Wasilewska A., Zalewska A. (2019). Salivary FRAP as a marker of chronic kidney disease progression in children. Antioxidants.

[153] Verma M.K., Jaiswal A., Sharma P., Kumar P., Singh A.N. (2019). Oxidative stress and biomarker of TNF-α, MDA and FRAP in hypertension. J. Med. Life.

[154] Tomandlova M., Parenica J., Lokaj P., Ondrus T., Kala P., Miklikova M., Helanova K., Helan M., Malaska J., Benesova K. (2021). Prognostic value of oxidative stress in patients with acute myocardial infarction complicated by cardiogenic shock: A prospective cohort study. Free Radic. Biol. Med..

[155] Wayner D.D.M., Burton G.W., Ingold K.U., Locke S. (1985). Quantitative measurement of the total peroxyl radical-trapping antioxidantcapability of human blood plasma by controlled peroxidation. FEBS Lett..

[156] Lissi E., Salim-Hanna M., Pascual C., DelCastillo M.D. (1995). Evaluation od total antioxidant potential (TRAP) and total antioxidant reactivity from luminol-enhanced chemiluminescence measurements. Free Radic. Biol. Med..

[157] Dresch M.T.K., Rossato S.B., Kappel V.D., Biegelmeyer R., Hoff M.L.M., Mayorga P., Zuanazzi J.A.S., Henriques A.T., Moreira J.C.F. (2009). Optimization and validation of an alternative method to evaluate total reactive antioxidant potential. Anal. Biochem..

[158] Praud D., Parpinel M., Serafini M., Belloco R., Tavani A., Lagiou P., LaVecchia C., Rossi M. (2015). Non-enzymatic antioxidant capacity and risk of gastric cancer. Cancer Epidemiol..

[159] Siegfried C.J., Shui Y.B. (2019). Intraocular oxygen and antioxidant status: New insights on the effect of vitrectomy and glaucoma pathogenesis. Am. J. Ophthalmol..

[160] Okimoto Y., Watanabe A., Niki E., Yamashita T., Noguchi N. (2000). A novel fluorescent probe diphenyl-1-pyrenylphosphine to follow lipid peroxidation in cell membranes. FEBS Lett..

[161] Takahashi M., Shibata M., Niki E. (2001). Estimation of lipid peroxidation od live cells using a fluorescent probe, Diphenyl-1-pyrenylphosphine. Free Radic. Biol. Med..

[162] Weinstein D.S., Liu W., Ngu K., Langevine C., Combs D.W., Zhuang S., Chen C., Madsen C.S., Harper T.W., Robl J.A. (2007). Discovery od selective imidazole-based inhibitors of mammalian 15-lipoxygenase: Highly potent against human enzyme within a cellular environment. Bioorg. Med. Chem. Lett..

[163] Dahlström M., Forsström D., Johannesson M., Huque-Andersson Y., Björk M., Silfverplatz E., Sanin A., Schaal W., Pelcman B., Forsell P.K.A. (2010). Development of a fluorescent intensity assay amenable for high throughput screening for determining 15-lipoxygenase activity. J. Biomol. Screen..

[164] Dobrian A.D., Lieb D.C., Cole B.K., Taylor-Fishwick D.A., Chakrabarti S.K., Nadler J.L. (2011). Functional and pathological roles of the 12- and 15-lipoxygenases. Prog. Lipid Res..

[165] Cropotova J., Rustad T. (2020). A new fluorimetric method for simultaneous determination of lipid and protein hydroperoxides in muscle foods with the use of diphenyl-1-pyrenylphosphine (DPPP). LTW Food Sci. Technol..

[166] Bligh E.G., Dyer W.J. (1959). A rapid method of total lipid extraction and purification. Can. J. Biochem. Physiol..

[167] Bou R., Chen B., Guardiola F., Codony R., Decker E.A. (2010). Determination of lipid and protein hydroperoxides using the fluorescent probe diphenyl-1-pyrenylphosphine. Food Chem..

[168] Zhou D., Shao L., Spitz D.R. (2014). Reactive oxygen species in normal and tumor stem cells. Adv. Cancer Res..

[169] Mirończuk-Chodakowska I., Witkowska A.M., Zujko M.E. (2018). Endogenous non-enzymatic antioxidants in the human body. Adv. Med. Sci..

[170] Dontha S. (2016). A review on antioxidant methods. Asian J. Pharm. Clin. Res..

[171] Rees K.R., Sinha K.P. (1960). Blood enzymes in liver injury. J. Pathol. Bacteriol..

[172] Townsend D.M., Tew K.D., Tapiero H. (2003). The importance of glutathione in human disease. Biomed. Pharm..

[173] Flohé L. (2013). The fairytale of the GSSG/GSH redox potential. Cell. Funct. Glutathione.

[174] Alli J.A., Kehinde A.O., Kosoko A.M., Ademowo O.G. (2014). Oxidative Stress and Reduced Vitamins C and E Levels Are Associated with Multi-Drug Resistant Tuberculosis. J. Tuberc. Res..

[175] Apostolova N., Victor V.M. (2015). Molecular strategies for targeting antioxidants to mitochondria: Therapeutic implications. Antioxid. Redox Signal..

[176] Chatterjee A. (2013). Reduced glutathione: A radioprotector or a modulator of DNA-repair activity. Nutrients.

[177] Polefka T.G., Meyer T.A., Agin P.P., Bianchini R.J. (2012). Cutaneous oxidative stress. J. Cosmet. Dermatol..

[178] Meng D., Zhang P., Zhang L., Wang H., Ho C.T., Li S., Shahidi F., Zhao H. (2017). Detection of cellular redox reactions and antioxidant activity assays. J. Funct. Foods.

[179] Brehe J.E., Burch H.B. (1976). Enzymatic assay for glutathione. Anal. Biochem..

[180] Liu J., Jia L., Kan J., Jin C. (2013). In vitro and in vivo antioxidant activity of ethanolic extract of white button mushroom (*Agaricus bisporus)*. Food Chem. Toxicol..

[181] Brigelius-Flohé R., Maiorino M. (2013). Glutathione peroxidases. Cell. Funct. Glutathione.

[182] Ye Z.W., Zhang J., Townsend D.M., Tew K.D. (2015). Oxidative stress, redox regulation and diseases of cellular differentiation. Redox Regul. Differ..

[183] Flohe L., Günzler W.A., Schock H.H. (1973). Glutathione peroxidase: A selenoenzyme. FEBS Lett..

[184] Thomson C.D. (2004). Assessment of requirements for selenium and adequacy of selenium status: A review. Eur. J. Clin. Nutr..

[185] Catarino M.D., Amarante S.J., Mateus N., Silva A.M.S., Cardoso S.M. (2021). Brown Algae Phlorotannins: A Marine Alternative to Break the Oxidative Stress, Inflammation and Cancer Network. Foods.

[186] Yang X., Yang S., Guo Y., Jiao Y., Zhao Y. (2013). Compositional characterization of soluble apple polysaccharides, and their antioxidant and hepatoprotective effects on acute CCl4-caused liver damage in mice. Food Chem..

[187] Strauss R.G., Snyder E.L., Wallace P.D., Rosenberger T.G. (1980). Oxygen-detoxifying enzymes in neutrophils of infants and their mothers. J. Lab. Clin. Med..

[188] Limón-Pacheco J., Gonsebatt M.E. (2009). The role of antioxidants and antioxidant-related enzymes in protective responses to environmentally induced oxidative stress. Oxidative Stress Mech. Environ. Toxic..

[189] Lewandowski Ł., Kepinska M., Milnerowicz H. (2020). Alterations in Concentration/Activity of Superoxide Dismutases in Context of Obesity and Selected Single Nucleotide Polymorphisms in Genes: SOD1, SOD2, SOD3. Int. J. Mol. Sci..

[190] Marklund S.L., Holme E., Hellner L. (1982). Superoxide dismutase in extracellular fluids. Clin. Chim. Acta Int. J. Clin. Chem..

[191] Yasui K., Kobayashi N., Yamazaki T., Agematsu K., Matsuzaki S., Ito S., Nakata S., Baba A., Koike K. (2005). Superoxide dismutase (SOD) as a potential inhibitory mediator of inflammation via neutrophil apoptosis. Free Radic. Res..

[192] Rondanelli M., Miraglia N., Putignano P., Castagliuolo I., Brun P., Dall’Acqua S., Peroni G., Faliva M.A., Naso M., Nichetti M. (2021). Effects of 60-day *Saccharomyces boulardii* and Superoxide Dismutase Supplementation on Body Composition, Hunger Sensation, Pro/Antioxidant Ratio, Inflammation and Hormonal Lipo-Metabolic Biomarkers in Obese Adultos: A Double-Blind, Placebo-Controlled Trial. Nutrients.

[193] Vaneev A.N., Kost O.A., Eremeev N.L., Beznos O.V., Alova A.V., Gorelkin P.V., Erofeev A.S., Chesnokova N.B., Kabanov A.V., Klyachko N.L. (2021). Superoxide Dismutase 1 Nanoparticles (Nano-SOD1) como um medicamento potencial para o tratamento de doenças inflamatórias dos olhos. Biomedicines.

[194] Rosa A.C., Corsi D., Cavi N., Bruni N., Dosio F. (2021). Superoxide Dismutase Administration: A Review of Proposed Human Uses. Molecules.

[195] Sgouros D., Katoulis A., Rigopoulos D. (2017). Novel topical agent containing superoxide dismutase 100 000 IU and 4% of plant extracts as a mono-therapy for atopic dermatitis. J. Cosmet. Dermatol..

[196] Bonetta R. (2018). Potential Therapeutic Applications of MnSODs and SOD-Mimetics. Chemistry.

[197] Chatterjee A., Zhu Y., Tong Q., Kosmacek E.A., Lichter E.Z., Oberley-Deegan R.E. (2018). The Addition of Manganese Porphyrins during Radiation Inhibits Prostate Cancer Growth and Simultaneously Protects Normal Prostate Tissue from Radiation Damage. Antioxidants.

[198] Heer C.D., Davis A.B., Riffe D.B., Wagner B.A., Falls K.C., Allen B.G., Buettner G.R., Beardsley R.A., Riley D.P., Spitz D.R. (2018). Superoxide Dismutase Mimetic GC4419 Enhances the Oxidation of Pharmacological Ascorbate and Its Anticancer Effects in an H₂O₂-Dependent Manner. Antioxidants.

[199] Coudriet G.M., Delmastro-Greenwood M.M., Previte D.M., Marré M.L., O’Connor E.C., Novak E.A., Vincent G., Mollen K.P., Lee S., Dong H.H. (2017). Treatment with a Catalytic Superoxide Dismutase (SOD) Mimetic Improves Liver Steatosis, Insulin Sensitivity, and Inflammation in Obesity-Induced Type 2 Diabetes. Antioxidants.

[200] Cline J.M., Dugan G., Bourland J.D., Perry D.L., Stitzel J.D., Weaver A.A., Jiang C., Tovmasyan A., Owzar K., Spasojevic I. (2018). Post-Irradiation Treatment with a Superoxide Dismutase Mimic, MnTnHex-2-PyP^5+^, Mitigates Radiation Injury in the Lungs of Non-Human Primates after Whole-Thorax Exposure to Ionizing Radiation. Antioxidants.

[201] Shrishrimal S., Kosmacek E., Chatterjee A., Tyson M., Oberley-Deegan R. (2017). The SOD Mimic, MnTE-2-PyP, Protects from Chronic Fibrosis and Inflammation in Irradiated Normal Pelvic Tissues. Antioxidantes.

[202] Wang J., Li J., Peng K., Fu Z.-Y., Tang J., Yang M.J., Chen Q.C. (2017). Association of the C47T polymorphism in superoxide dismutase gene 2 with noise-induced hearing loss: A meta-analysis. Braz. J. Otorhinolaryngol..

[203] Abati E., Bresolin N., Comi G., Corti S. (2020). Silence superoxide dismutase 1 (SOD1): A promising therapeutic target for amyotrophic lateral sclerosis (ALS). Expert Opin. Ther. Targets.

[204] Brigelius-Flohé R., Flohé L. (2011). Basic principles and emerging concepts in the redox control of transcription factors. Antioxid. Redox Signal..

[205] Mehmet A., Hilal A., Dilek O., Ferruh A., Abdullah G., Murat C., Serhan Y., Volkan H., Gamze K., Işıl T. (2015). Effects of various anesthesia maintenance on serum levels of selenium, copper, zinc, iron and antioxidant capacity. Braz. J. Anesthesiol..

[206] Collins Y., Chouchani E.T., James A.M., Menger K.E., Cochemé H.M., Murphy M.P. (2012). Mitochondrial redox signalling at a glance. J. Cell Sci..

[207] Mittal M., Siddiqui M.R., Tran K., Reddy S.P., Malik A.B. (2014). Reactive oxygen species in inflammation and tissue injury. Antioxid. Redox Signal..

[208] Rodrigues G.P., Cozzolino S.F., Marreiro D.N., Caldas D.C., Silva K.G., Sousa Almondes K.G., Neto J.M., Pimentel J.C., de Carvalho C.G., Nogueira N.N. (2017). Mineral status and superoxide dismutase enzyme activity in Alzheimer’s disease. J. Trace Elem. Med. Biol..

[209] Halliwell B., Gutteridge J.M. (1990). Role of free radicals and catalytic metal ions in human disease: An overview. Methods Enzymol..

[210] Griess B., Tom E., Domann F., Teoh-Fitzgerald M. (2017). Extracellular superoxide dismutase and its role in cancer. Free Radic. Biol. Med..

[211] Quick K.L., Hardt J.I., Dugan L.L. (2000). Rapid microplate assay for superoxide scavenging efficiency. J. Neurosci. Methods.

[212] Yamashita N., Murata M., Inoue S., Burkitt M.J., Milne L., Kawanishi S. (1998). Alpha-tocopherol induces oxidative damage to DNA in the presence of copper (II) ions. Chem. Res. Toxicol..

[213] Chelikani P., Fita I., Loewen P.C. (2004). Diversity of structures and properties among catalases. Cell. Mol. Life Sci. CMLS.

[214] Gaetani G.F., Ferraris A.M., Rolfo M., Mangerini R., Arena S., Kirkman H.N. (1996). Predominant role of catalase in the disposal of hydrogen peroxide within human erythrocytes. Blood.

[215] Lobo V., Patil A., Phatak A., Chandra N. (2010). Free radicals, antioxidants and functional foods: Impact on human health. Pharmacogn. Ver..

[216] Ighodaro O.M., Akinloye O.A. (2017). First line defence antioxidants-superoxide dismutase (SOD), catalase (CAT) and glutathione peroxidase (GPX): Their fundamental role in the entire antioxidant defence grid. Alex. J. Med..

[217] Eisner T., Aneshansley D.J. (1999). Spray aiming in the bombardier beetle: Photographic evidence. Proc. Natl. Acad. Sci. USA.

[218] Glorieux C., Calderon P.B. (2017). Catalase, a remarkable enzyme: Targeting the oldest antioxidant enzyme to find a new cancer treatment approach. Biol Chem..

[219] Hadwan M.H. (2018). Ensaio espectrofotométrico simples para medir a atividade da catalase em tecidos biológicos. BMC Biochem..

[220] Ceci R., Duranti G., Leonetti A., Pietropaoli S., Spinozzi F., Marcocci L., Amendola R., Cecconi F., Sabatini S., Mariottini P. (2017). Adaptive responses of heart and skeletal muscle to spermine oxidase overexpression: Evaluation of a new transgenic mouse model. Free Radic. Biol. Med..

[221] Nelson S.K., Bose S.K., Grunwald G.K., Myhill P., McCord J.M. (2006). The induction of human superoxide dismutase and catalase in vivo: A fundamentally new approach to antioxidant therapy. Free Radic. Biol. Med..

[222] Forman H.J., Augusto O., Brigelius-Flohe R., Dennery P.A., Kalyanaraman B., Ischiropoulos H., Mann G.E., Radi R., Roberts L.J., Vina J. (2015). Even free radicals should follow some rules: A Guide to free radical research terminology and methodology. Free Radic. Biol. Med..

[223] Kadiiska M.B., Gladen B.C., Baird D.D., Germolec D., Graham L.B., Parker C.E., Nyska A., Wachsman J.T., Ames B.N., Basu S. (2005). Biomarkers of Oxidative Stress Study II: Are oxidation products of lipids, proteins, and DNA markers of CCl4 poisoning. Free Radic. Biol. Med..

[224] Draijer R., Van Dorsten F.A., Zebregs Y.E., Hollebrands B., Peters S., Duchateau G.S., Grün C.H. (2016). Impact of Proteins on the Uptake, Distribution, and Excreetion of Phenolics in the Human Body. Nutrients.

[225] Parada J., Aguilera J.M. (2007). Food microstructure affects the bioavailability of several nutrients. J. Food Sci..

[226] Kasprzak-Drozd K., Oniszczuk T., Stasiak M., Oniszczuk A. (2021). Beneficial Effects of Phenolic Compounds on Gut Microbiota and Metabolic Syndrome. Int. J. Mol. Sci..

[227] Pérez-Jiménez J., Serrano J., Tabernero M., Arranz S., Díaz-Rubio M.E., García-Diz L., Goñi I., Saura-Calixto F. (2009). Bioavailability of phenolic antioxidants associated with dietary fiber: Plasma antioxidant capacity after acute and long-term intake in humans. Plant Foods Hum. Nutr..

[228] Williamson G., Clifford M.N. (2017). Effect of thermal processing on free and bound phenolic compounds and antioxidant activities of hawthorn. Biochem. Pharmacol..

[229] Del Rio D., Costa L., Lean M., Crozier A. (2010). Polyphenols and health: What compounds are involved. Nutr. Metab. Cardiovasc. Dis..

[230] Kawai Y. (2018). Understanding metabolic conversions and molecular actions of flavonoids in vivo: Toward new strategies for effective utilization of natural polyphenols in human health. J. Med. Investig..

[231] Kumar N., Goel N. (2019). Phenolic acids: Natural versatile molecules with promising therapeutic applications. Biotechnol. Rep..

[232] Palafox-Carlos H., Ayala-Zavala J.F., González-Aguilar G.A. (2011). The Role of Dietary Fiber in the Bioaccessibility and Bioavailability of Fruit and Vegetable Antioxidants. J. Food Sci..

[233] Lafay S., Gil-Izquierdo A. (2008). Bioavailability of phenolic acids. Phytochem. Rev..

[234] Lafay S., Gil-Izquierdo A., Manach C., Morand C., Besson C., Scalbert A. (2006). Chlorogenic acid is absorbed in its intact form in the stomach of rats. J. Nutr..

[235] Yonekura L., Martins C.A., Sampaio G.R., Monteiro M.P., César L.M., Mioto B.M., Mori C.S., Mendes T.M.N., Ribeiro M.L., Arçari D.P. (2016). Bioavailability of catechins from guaraná (*Paullinia cupana*) and its effect on antioxidant enzymes and other oxidative stress markers in healthy human subjects. Food Funct..

[236] Yao H.T., Hsu Y.R., Lii C.K., Lin A.H., Chang K.H., Yang H.T. (2014). Effect of commercially available green and black tea beverages on drug-metabolizing enzymes and oxidative stress in Wistar rats. Food Chem. Toxicol. Int. J. Publ. Br. Ind. Biol. Res. Assoc..

[237] Lara-Guzmán O.J., Álvarez-Quintero R., Osorio E., Naranjo-Cano M., Muñoz-Durango K. (2016). CGMS method to quantify bioavailable phenolic compounds and antioxidant capacity determination of plasma after acute coffee consumption in human volunteers. Food Res. Int. Ott. Ont..

[238] Yeh H., Chuang C., Chen H., Wan C., Chen T., Lin L. (2014). Bioactive components analysis of two various gingers (*Zingiber officinale* Roscoe) and antioxidant effect of ginger extracts. LWT Food Sci. Technol..

[239] Sauberlich H. (1985). Bioavailability of vitamins. Prog. Food Nutr. Sci..

[240] Naidu K.A. (2003). Vitamin C in human health and disease is still a mystery—An overview. Nutr. J..

[241] Borel P., Preveraud D., Desmarchelier C. (2013). Bioavailability of vitamin E in humans: An update. Nutr. Rev..

[242] Iwata N., Okazaki M., Xuan M., Kamiuchi S., Matsuzaki H., Hibino Y. (2014). Orally Administrated Ascorbic Acid Suppresses Neuronal Damage and Modifies Expression of SVCT2 and GLUT1 in the Brain of Diabetic Rats with Cerebral Ischemia-Reperfusion. Nutrients.

[243] Bayrak B.B., Çatal T., Öztay F., Yanardağ R., Bolkent Ş. (2016). Efficacy of antioxidant vitamins (vitamin C, vitamin E, beta-carotene) and selenium supplement on D-galactosamine-induced lung injury. IUFS J. Biol..

[244] Catal T., Tunali S., Bolkent S., Yanardag R. (2017). An Antioxidant Combination Improves Histopathological Alterations and Biochemical Parameters in D-Galactosamine-Induced Hepatotoxicity in Rats. Eur. J. Biol..

[245] Donhowe E.G., Kong F. (2014). Beta-carotene: Digestion, microencapsulation, and in vitro bioavailability. Food Bioprocess Technol..

[246] Desmarchelier C., Borel P. (2017). Overview of carotenoid bioavailability determinants: From dietary factors to host genetic variations. Trends Food Sci. Technol..

[247] Bohn T., Desmarchelier C., Dragsted L.O., Nielsen C.S., Stahl W., Rühl R., Keijer J., Borel P. (2017). Host-related factors explaining interindividual variability of carotenoid bioavailability and tissue concentrations in humans. Mol. Nutr. Food Res..

[248] Ho N., Inbaraj B.S., Chen B.H. (2016). Utilization of microemulsions from *Rhinacanthus nasutus* (L.) Kurz to improve carotenoid bioavailability. Sci. Rep..

[249] Aschoff J.K., Rolke C.L., Breusing N., Bosy-Westphal A., Högel J., Carle R., Schweiggert R.M. (2015). Bioavailability of β-cryptoxanthin is greater from pasteurized orange juice than from fresh oranges–a randomized cross-over study. Mol. Nutr. Food Res..

[250] Van het Hof K.H., West C.E., Weststrate J.A., Hautvast J.A.J. (2000). Dietary Factors That Affect the Bioavailability of Carotenoids. J. Nutr..

[251] Gupta R., Kopec R.E., Schwartz S.J., Balasubramaniam V. (2011). Combined pressure–temperature effects on carotenoid retention and bioaccessibility in tomato juice. J. Agric. Food Chem..

[252] Buniowska M., Carbonell-Capella J.M., Frigola A., Esteve M.J. (2017). Bioaccessibility of bioactive compounds after non-thermal processing of an exotic fruit juice blend sweetened with Stevia rebaudiana. Food Chem..

[253] Ranga Rao A., Baskaran V., Sarada R., Ravishankar G.A. (2013). In vivo bioavailability and antioxidant activity of carotenoids from microalgal biomass—A repeated dose study. Food Res. Int..

[254] Ravi H., Baskaran V. (2017). Chitosan-glycolipid nanocarriers improve the bioavailability of fucoxanthin via up-regulation of PPARγ and SRB1 and antioxidant activity in rat model. J. Funct. Foods.

[255] Campos K.K.D., Araújo G.R., Martins T.L., Bandeira A.C.B., Costa G.P., Talvani A., Garcia C.C.M., Oliveira L.A.M., Costa D.C., Bezerra F.S. (2017). The antioxidant and anti-inflammatory properties of lycopene in mice lungs exposed to cigarette smoke. J. Nutr. Biochem..

[256] Gökmen V., Serpen A., Fogliano V. (2009). Direct measurement of the total antioxidant capacity of foods: The ‘QUENCHER’ approach. Trends Food Sci. Technol..

[257] Del Pino-García R., García-Lomillo J., Rivero-Pérez M.D., González-SanJosé M.L., Muñiz P. (2015). Adaptation and Validation of QUick, Easy, New, CHEap, and Reproducible (QUENCHER) Antioxidant Capacity Assays in Model Products Obtained from Residual Wine Pomace. J. Agric. Food Chem..

[258] Kitrytė V., Šaduikis A., Venskutonis P.R. (2015). Assessment of antioxidant capacity of brewer’s spent grain and its supercritical carbon dioxide extract as sources of valuable dietary ingredients. J. Food Eng..

[259] Laus M.N., Di Benedetto N.A., Caporizzi R., Tozzi D., Soccio M., Giuzio L., De Vita P., Flagella Z., Pastore D. (2015). Evaluation of Phenolic Antioxidant Capacity in Grains of Modern and Old Durum Wheat Genotypes by the Novel QUENCHERABTS Approach. Plant Foods Hum. Nutr. Dordr. Neth..

[260] Shahbaz H.M., Park E.J., Kim G.R., Akram K., Kwon J.H. (2016). Assessment of antioxidant potential of pomegranate fruit by-products via a direct approach using a simple quencher method. J. AOAC Int..

[261] Keller J.N., Kindy M.S., Holtsberg F.W., Clair D.K.S., Yen H.C., Germeyer A., Steiner S.M., Bruce-Keller A.J., Hutchins J.B., Mattson M.P. (1998). Mitochondrial manganese superoxide dismutase prevents neural apoptosis and reduces ischemic brain injury: Suppression of peroxynitrite production, lipid peroxidation, and mitochondrial dysfunction. J. Neurosci. Off. J. Soc. Neurosci..

[262] Wang P., Chen H., Qin H., Sankarapandi S., Becher M.W., Wong P.C., Zweier J.L. (1998). Overexpression of human copper, zinc-superoxide dismutase (SOD1) prevents postischemic injury. Proc. Natl. Acad. Sci. USA.

[263] Fujita H., Fujishima H., Chida S., Takahashi K., Qi Z., Kanetsuna Y., Breyer M.D., Harris R.C., Yamada Y., Takahashi T. (2009). Reduction of renal superoxide dismutase in progressive diabetic nephropathy. J. Am. Soc. Nephrol. JASN.

[264] Nguyen C.T., Sah S.K., Zouboulis C.C., Kim T.Y. (2018). Inhibitory effects of superoxide dismutase 3 on Propionibacterium acnes-induced skin inflammation. Sci Rep..

[265] Agrahari G., Sah S.K., Nguyen C.T., Choi S.S., Kim H.Y., Kim T.Y. (2020). Superoxide Dismutase 3 Inhibits LL-37/KLK-5-Mediated Skin Inflammation through Modulation of EGFR and Associated Inflammatory Cascades. J. Investig. Dermatol..

[266] Groner Y., Elroy-Stein O., Bernstein Y., Dafni N., Levanon D., Danciger E., Neer A. (1986). Molecular genetics of Down’s syndrome: Overexpression of transfected human Cu/Zn-superoxide dismutase gene and the consequent physiological changes. Cold Spring Harb. Symp. Quant. Biol..

[267] Amstad P., Peskin A., Shah G., Mirault M.E., Moret R., Zbinden I., Cerutti P. (1991). The balance between copper-zinc superoxide dismutase and catalase affects the sensitivity of mouse epidermal cells to oxidative stress. Biochemistry.

[268] Amstad P., Moret R., Cerutti P. (1994). Glutathione peroxidase compensates for the hypersensitivity of Cu, Zn-superoxide dismutase overproducers to oxidant stress. J. Biol. Chem..

[269] Kowald A., Lehrach H., Klipp E. (2006). Alternative pathways as mechanism for the negative effects associated with overexpression of superoxide dismutase. J. Theor. Biol..

[270] Sinet P.M. (1982). Metabolism of oxygen derivatives in Down’s syndrome. Ann. N. Y. Acad. Sci..

[271] Abaspour A.R., Taghikhani M., Parizade M.R., Moohebati M., Ghafoori F., Mehramiz M., Tayefi M., Avan A., Ghalandari M., Ferns G.A.A. (2017). Association between Serum Glutathione Peroxidases and Superoxide Dismutases mRNA Level with Coronary Artery Disease. Health.

[272] Salem K., McCormick M.L., Wendlandt E., Zhan F., Goel A. (2014). Copper–zinc superoxide dismutase-mediated redox regulation of bortezomib resistance in multiple myeloma. Redox Biol..

[273] Glorieux C., Zamocky M., Sandoval J.M., Verrax J., Calderon P.B. (2015). Regulation of catalase expression in healthy and cancerous cells. Free Radic. Biol. Med..

[274] Röhrdanz E., Schmuck G., Ohler S., Kahl R. (2001). The influence of oxidative stress on catalase and MnSOD gene transcription in astrocytes. Brain Res..

[275] Röhrdanz E., Kahl R. (1998). Alterations of antioxidant enzyme expression in response to hydrogen peroxide. Free Radic. Biol. Med..

[276] Sen P., Mukherjee S., Bhaumik G., Das P., Ganguly S., Choudhury N., Raha S. (2003). Enhancement of catalase activity by repetitive low-grade H2O2 exposures protects fibroblasts from subsequent stress-induced apoptosis. Mutat. Res..

[277] Giustarini D., Tsikas D., Colombo G., Milzani A., Dalle-Donne I., Fanti P., Rossi R. (2016). Pitfalls in the analysis of the physiological antioxidant glutathione (GSH) and its disulfide (GSSG) in biological samples: An elephant in the room. Anal. Tools Protoc. Oxidative Stress.

[278] Hedrén E., Diaz V., Svanberg U. (2002). Estimation of carotenoid accessibility from carrots determined by an in vitro digestion method. Eur. J. Clin. Nutr..

[279] Mason S.A., Della Gatta P.A., Snow R.J., Russell A.P., Wadley G.D. (2016). Ascorbic acid supplementation improves skeletal muscle oxidative stress and insulin sensitivity in people with type 2 diabetes: Findings of a randomized controlled study. Free Radic. Biol. Med..

[280] Kagan V.E., Tyurina Y.Y. (2006). Recycling and Redox Cycling of Phenolic Antioxidants. Ann. N. Y. Acad. Sci..

[281] Bayir H., Kagan V.E., Tyurina Y.Y., Tyurin V., Ruppel R.A., Adelson P.D., Graham S.H., Janesko K., Clark R.S.B., Kochanek P.M. (2002). Assessment of Antioxidant Reserves and Oxidative Stress in Cerebrospinal Fluid after Severe Traumatic Brain Injury in Infants and Children. Pediatr. Res..

[282] Bast A., Haenen G.R.M. (2013). Ten misconceptions about antioxidants. Trends Pharmacol. Sci..

[283] Heaney R.P. (2008). Nutrients, endpoints, and the problem of proof. J. Nutr..

